# HNRNPU K181 Lactylation Drives Cervical Cancer Growth by Upregulating PHGDH and Reprogramming Serine Metabolism

**DOI:** 10.1002/advs.202520222

**Published:** 2026-05-27

**Authors:** Chang Zhang, Qingfei Meng, Hui Jiao, Huixin Liu, Xiangchao Wang, Honglan Zhou, Yishu Wang

**Affiliations:** ^1^ Key Laboratory of Pathobiology Ministry of Education Jilin University Changchun China; ^2^ Department of Urology The First Hospital of Jilin University Changchun China

**Keywords:** cervical cancer, HNRNPU, lactylation, PHGDH, RNA binding, Serine metabolism

## Abstract

Cervical cancer remains a major global health burden, yet the molecular mechanisms driving its metabolic reprogramming are incompletely understood. Here, we identify heterogeneous nuclear ribonucleoprotein U (HNRNPU) as a novel non‐histone substrate of lysine lactylation that links lactate accumulation to serine metabolism and tumor progression. We demonstrate that lactylation at lysine 181 (K181) stabilizes HNRNPU, enhances its binding to PHGDH mRNA, and maintains the exon 1‐containing PHGDH transcript and mRNA stability, thereby sustaining PHGDH expression and activating the serine biosynthesis pathway. This metabolic rewiring promotes redox homeostasis, nucleotide synthesis, and cervical cancer cell proliferation both in vitro and in vivo. Mechanistically, we reveal a competitive interplay between lactylation and NAA50‐mediated acetylation at K181, establishing a dynamic post‐translational modification switch that fine‐tunes HNRNPU function. Importantly, pharmacological inhibition of HNRNPU K181 lactylation by Pazopanib suppresses PHGDH expression and tumor growth, underscoring its translational potential. Collectively, our findings uncover a lactate‐driven regulatory axis in which HNRNPU K181 lactylation integrates metabolic signaling with post‐transcriptional regulation to promote cervical cancer progression, providing a promising therapeutic avenue for targeting metabolic vulnerabilities in malignancies.

## Graphical abstract

Lactate in cervical cancer induces HNRNPU K181 lactylation, opposed by NAA50‐mediated acetylation and suppressed by Pazopanib. This lactylation enhances HNRNPU binding to PHGDH pre‐mRNA exon 1, maintaining exon 1‐containing transcripts and mRNA stability, thereby activating serine metabolism. HNRNPU K181 mutation disrupts these effects, reducing PHGDH expression and cancer progression.

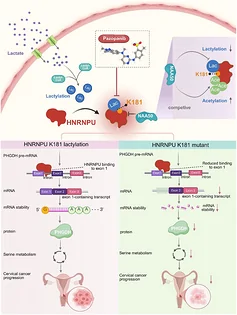

## 1 Introduction

Cervical cancer ranks as the fourth most common malignancy among women worldwide, and its incidence has been on the rise in recent years [1, 2, 3, 4]. Although significant progress has been made in the diagnosis and treatment of cervical cancer, the molecular mechanisms underlying its initiation and progression remain incompletely understood. Metabolic reprogramming has been recognized as a key hallmark of cancer [5, 6]. Lactate, a terminal product of glycolysis, is abundantly accumulated in rapidly proliferating tumor cells due to the Warburg effect [7]. Extensive studies have demonstrated that lactate not only promotes cancer cell proliferation [8] but also contributes to chemoresistance [9] and the formation of a pro‐tumor microenvironment [10]. More recently, lactylation has been identified as a novel post‐translational modification (PTM) of lysine residues on both histone and non‐histone proteins, providing new insights into the non‐metabolic functions of lactate [11]. Histone lactylation has been shown to play essential roles in regulating macrophage polarization and the reprogramming of embryonic fibroblasts into pluripotent stem cells [12]. In parallel, non‐histone protein lactylation has attracted increasing attention due to its potential involvement in cancer progression. For instance, lactylation of DCBLD1 has been reported to enhance cervical cancer cell proliferation and metastasis [13]. Despite these advances, the regulatory landscape of protein lactylation in cervical cancer remains poorly defined, and its functional relevance requires further investigation.

Heterogeneous nuclear ribonucleoprotein U (HNRNPU) is a multifunctional RNA‐binding protein involved in post‐transcriptional regulation, chromatin organization, and epigenetic modulation via interactions with non‐coding RNAs [14]. Its N‐terminal SAP domain mediates binding to scaffold/matrix attachment regions (S/MARs), while its C‐terminal RGG‐rich region confers specificity in interacting with various mRNAs and long non‐coding RNAs, such as Xist and NEAT1 [15, 16]. HNRNPU has been implicated in alternative splicing, RNA stability, and nuclear export (Heterogeneous Nuclear Ribonucleoproteins Involved in the Functioning of Telomeres in Malignant Cells). Aberrant expression of HNRNPU has been observed in multiple cancers, where it modulates oncogenic RNA networks and signaling pathways to promote tumor cell proliferation, migration, and transcriptional plasticity [17, 18]. Given the emerging role of lysine lactylation as a non‐histone PTM, HNRNPU has been proposed as a potential lactylation substrate or modulator, mediating cancer cell responses to metabolic changes. However, whether HNRNPU undergoes lactylation in cervical cancer, and how this modification influences its functional roles in tumor progression, remains largely unexplored.

Serine metabolism plays a critical role in supporting the rapid proliferation and epigenetic regulation of cancer cells [19]. The serine synthesis pathway (SSP), derived from the glycolytic intermediate 3‐phosphoglycerate, is catalyzed by three key enzymes: Phosphoglycerate Dehydrogenase (PHGDH), Phosphoserine Aminotransferase 1 (PSAT1), and Phosphoserine Phosphatase (PSPH). This pathway provides one‐carbon units essential for nucleotide synthesis and methylation processes [20]. This pathway can be transcriptionally activated by oncogenic signals such as c‐Myc and ATF4, enhancing metabolic plasticity under stress conditions [21, 22]. In addition to its metabolic role, lactate has been shown to exert non‐metabolic effects by regulating enzymatic activity and nuclear protein function through lactylation. Given HNRNPU's central role in RNA splicing and gene regulation, its potential lactylation may influence the post‐transcriptional expression of key metabolic enzymes, such as PHGDH, thereby contributing to metabolic reprogramming in cervical cancer cells.

In this study, we report for the first time that HNRNPU functions as a lactylation substrate linking serine metabolism to alternative splicing in cervical cancer, ultimately promoting tumor cell proliferation. Specifically, under high‐lactate conditions, the acetyltransferase NAA50 was found to associate with HNRNPU at lysine 181 (K181), mediating a competitive interplay between acetylation and lactylation at this site. Functional analyses revealed that HNRNPU directly binds PHGDH mRNA, and that the K181A mutation reduces the exon 1‐containing PHGDH transcript and decreases PHGDH mRNA stability. This leads to suppression of the serine biosynthesis pathway. Our findings delineate a novel mechanism by which lactate modulates HNRNPU function in cervical cancer and suggest that lactylation at K181 (K181lac) may serve as a potential therapeutic target for metabolic intervention.

## 2 Results

### 2.1 HNRNPU Promotes Cervical Cancer Cell Growth

**FIGURE 1:**
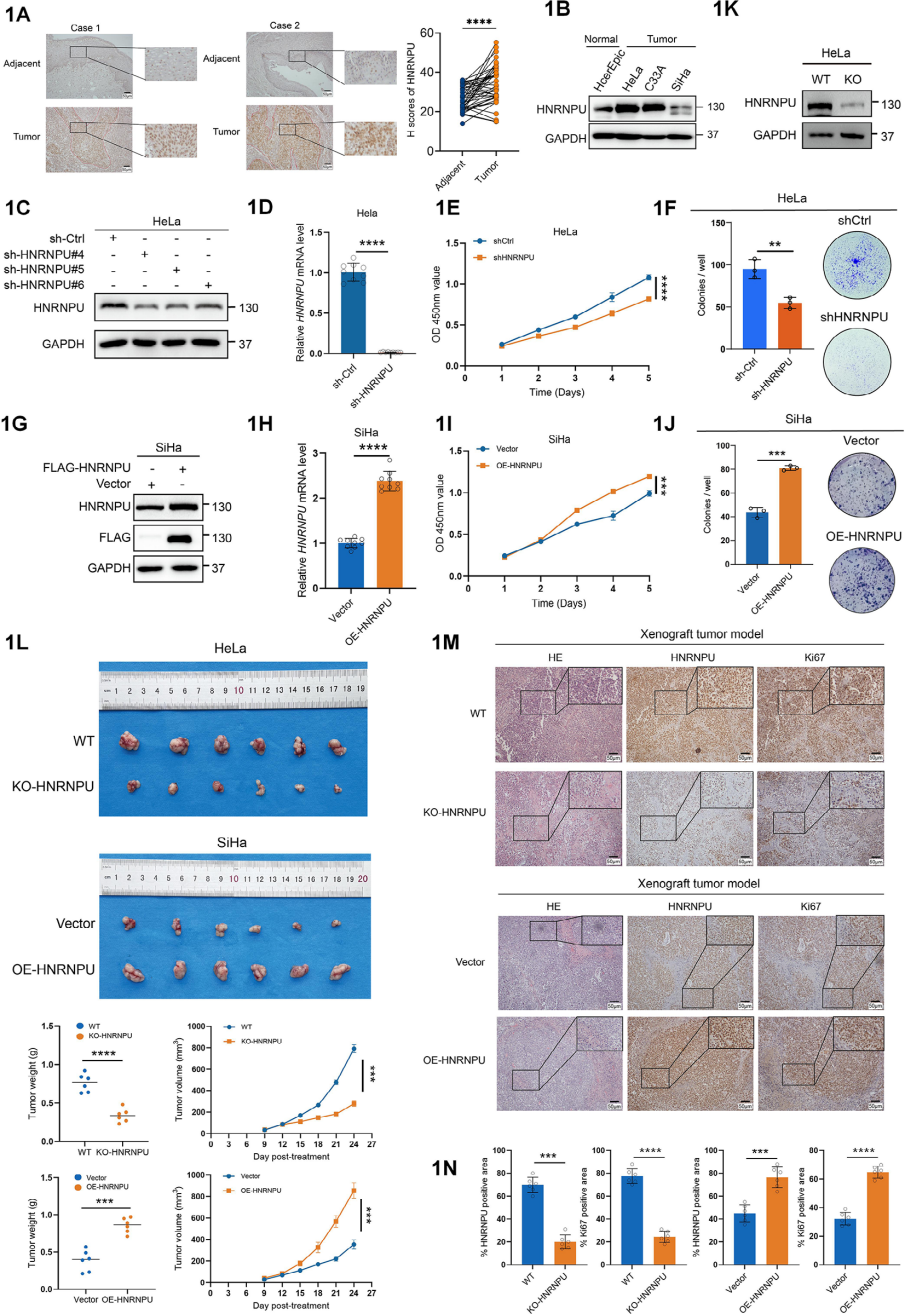
HNRNPU promotes cervical cancer cell proliferation both in vitro and in vivo. (A) Representative IHC staining of HNRNPU in human cervical cancer tissues and adjacent normal tissues. Scale bar: 50 µm. Dotted lines delineate the boundary between tumor and stroma. Scatter plot comparing HNRNPU H‐scores in tumor tissues versus matched adjacent normal tissues. Each data point represents one sample; lines connect paired tissues from the same patient (*n* = 45 pairs). Statistical significance was assessed by paired‐samples *t*‐test, ****p < 0.0001. (B) Western blot analysis of HNRNPU expression in normal cervical epithelial cells (HcerEpic) and cervical cancer cell lines (HeLa, C33A, SiHa). (Original uncropped blots can be found in Supporting Information S2: “Original blots for Figure 1B.”) (C) Western blot analysis of HNRNPU expression in HeLa cells transfected with control shRNA (sh‐Ctrl) or three independent shRNAs targeting HNRNPU (sh‐HNRNPU#4, #5, #6). Representative images from three independent biological replicates are shown. (Original uncropped blots can be found in Supporting Information S2: “Original blots for Figure 1C.”) (D) Relative mRNA expression levels of HNRNPU following knockdown. Data are presented as mean ± SD from three independent biological replicates, each with three technical replicates (*n* = 9 total data points shown). Individual data points are displayed. Statistical significance was determined using a two‐tailed unpaired t‐test. ****p < 0.0001. (E,F) Cell proliferation was assessed by CCK‐8 assay in HNRNPU‐knockdown HeLa cells. Data are presented as mean ± SD (*n* = 4 per group). Overall growth curves were analyzed using two‐way repeated‐measures ANOVA with group and time as factors. Statistical significance indicated by brackets was determined at the final time point (day 5) using a two‐tailed unpaired t‐test. ****p < 0.0001. Colony formation images are representative of three independent experiments, with quantification shown in the left panel. Data are presented as mean ± SD (*n* = 3). Statistical analysis was performed using two‐tailed unpaired t‐test. **p < 0.01. (G) Western blot analysis confirming HNRNPU overexpression in SiHa cells. Representative images from three independent biological replicates are shown. (Original uncropped blots can be found in Supporting Information S2: “Original blots for Figure 1G.”) (H) Relative mRNA expression levels of HNRNPU following expression. Data are presented as mean ± SD from three independent biological replicates, each with three technical replicates (*n* = 9 total data points shown). Individual data points are displayed. Statistical significance was determined using a two‐tailed unpaired t‐test. ****p < 0.0001. (I,J) Cell proliferation was assessed by CCK‐8 assay in HNRNPU‐overexpressing SiHa cells. Data are presented as mean ± SD (*n* = 4 per group). Overall growth curves were analyzed using two‐way repeated‐measures ANOVA with group and time as factors. Statistical significance indicated by brackets was determined at the final time point (day 5) using a two‐tailed unpaired t‐test. ***p < 0.001. Colony formation images are representative of three independent experiments, with quantification shown in the left panel. Data are presented as mean ± SD (*n* = 3). Statistical analysis was performed using two‐tailed unpaired t‐test, ***p < 0.001. (K) Immunoblot analysis confirming HNRNPU knockout in HeLa cells. Representative images from three independent biological replicates are shown. (Original uncropped blots can be found in Supporting Information S2: “Original blots for Figure 1K.”) (L) Xenograft tumor formation assay showing representative tumors, tumor growth curves, and excised tumor weights from mice implanted with control or HNRNPU‐modulated HeLa/SiHa cells (*n* = 6 per group). Tumor volumes were measured every 3 days and are presented as mean ± SD. Overall tumor growth curves were analyzed using two‐way repeated‐measures ANOVA with group and time as factors. Statistical significance indicated in the growth curves was determined at the final time point (day 24) using a two‐tailed unpaired t‐test. Tumor weights were analyzed using two‐tailed unpaired t‐test. ***p < 0.001, ****p < 0.0001. (M, N) Representative images of HE staining and IHC staining of HNRNPU and Ki67 in xenograft tumor sections from the indicated groups. Scale bar: 50 µm. Quantification of HNRNPU‐ and Ki67‐positive cells (percentage of positive cells) is shown in the lower panel. Data are presented as mean ± SD (*n* = 6 mice per group), with each data point representing one mouse. Statistical significance was determined by two‐tailed unpaired t‐test. ***p < 0.001, ****p < 0.0001.

In cervical cancer, aberrant alternative splicing is a critical post‐transcriptional regulatory mechanism that drives malignant phenotypes by generating pro‐oncogenic isoforms, thereby influencing cell proliferation, metabolism, and invasion [23]. Splicing factors, the core executors of splicing regulation, are frequently dysregulated in various cancers, closely linked to disease progression. Consequently, the systematic identification of key splicing factors in cervical cancer is essential for deciphering disease‐specific splicing networks and uncovering novel therapeutic targets. To this end, we first analyzed the expression profiles of known splicing factor families in cervical cancer using the GEPIA database. This revealed that members of the HNRNP family exhibited significantly higher overall expression levels compared to other families (Figure S1A i), suggesting their potential pivotal role in this malignancy. However, analysis of transcriptomic data from the TCGA‐CESC cohort showed no significant difference in mRNA levels of key HNRNP family members (including HNRNPU, HNRNPM, and HNRNPD) between tumor and normal tissues (Figure S1A ii). Given that HNRNPU, HNRNPM, and HNRNPD have been previously reported to possess well‐defined pro‐tumorigenic functions in various cancers, including gastric, colorectal, breast, and lung cancers, as well as osteosarcoma [14, 24, 25, 26, 27, 28, 29, 30], we hypothesized that their important roles in cervical cancer might be regulated at the post‐transcriptional or post‐translational level. To test this hypothesis, we analyzed data from the proteomics database PRIDE (PXD055203), which comprised 136 cervical cancer tissues and 33 normal tissues. The analysis demonstrated that the protein levels of HNRNPU, HNRNPM, and HNRNPD were significantly elevated in tumor tissues compared to normal tissues, a trend that was also consistent in paired samples (Figure S1B,C). To further evaluate their hub status within regulatory networks, we constructed a high‐confidence interaction network centered on these three proteins using the STRING database. Topological analysis indicated that HNRNPU occupied a central node position within this network. Its node degree, betweenness centrality, and closeness centrality were significantly higher than those of the other members, and it formed dense connections with multiple key splicing factors, RNA metabolism proteins, and signaling pathway molecules (Figure S1D). This demonstrates that HNRNPU may function as a core hub protein, integrating the splicing regulatory network in cervical cancer. Consistent with this bioinformatic prediction and the proteomics data, immunohistochemical (IHC) analysis of our clinical specimens confirmed a significant upregulation of HNRNPU at the protein level, with weak immunoreactivity observed in normal cervical tissues compared to strong staining in carcinoma samples (Figure 1A). Western blot analysis confirmed markedly higher HNRNPU protein expression in cervical cancer cell lines (HeLa and C33A) relative to normal cervical epithelial cells (HcerEpic) (Figure 1B). Functional characterization was performed through shRNA‐mediated knockdown in HeLa cells (Figure 1C,D), resulting in significantly reduced cell viability (Figure 1E) and colony formation capacity (Figure 1F). Conversely, HNRNPU overexpression in SiHa cells was found to enhance both proliferative potential and clonogenicity (Figures 1G–J). These in vitro findings were validated in vivo through xenograft models. CRISPR‐Cas9‐generated HNRNPU‐knockout HeLa cells (Figure 1K) formed significantly smaller tumors relative to controls, whereas HNRNPU‐overexpressing SiHa cells produced larger tumors (Figure 1L). Immunohistochemical analysis of proliferating cell nuclear antigen (Ki67) in xenograft tissues demonstrated reduced staining in HNRNPU‐knockout tumors and elevated expression in HNRNPU‐overexpressing tumors (Figure 1M,N), further supporting these observations.

### 2.2 L‐Lactate Stabilizes HNRNPU Through Direct Lactylation Modifications

**FIGURE 2:**
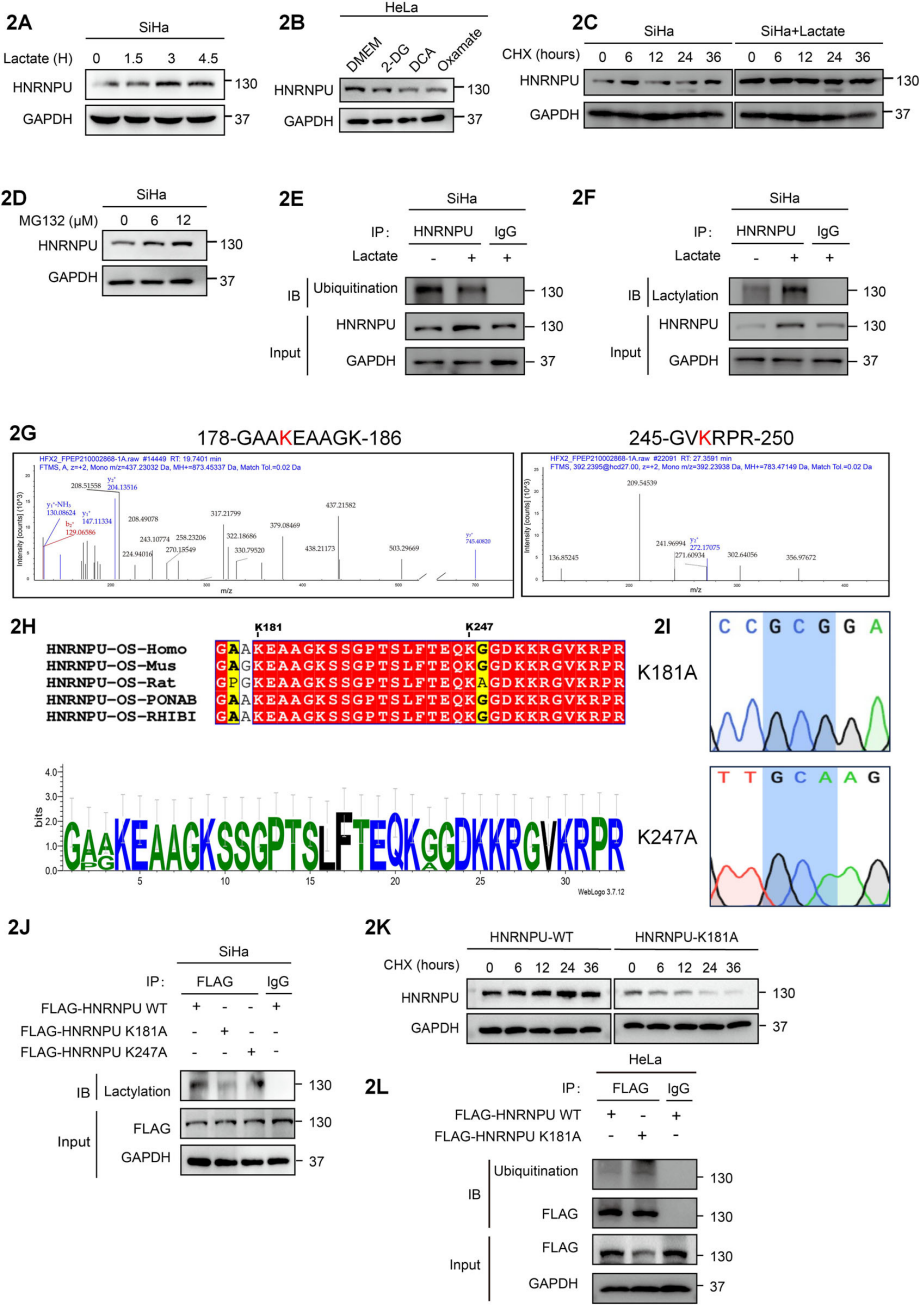
Lactylation stabilizes HNRNPU expression by modulating HNRNPU ubiquitination and degradation. (A) Time‐dependent effects of sodium L‐lactate (25 mM) on HNRNPU protein levels in SiHa cells, assessed by western blotting. Representative images from three independent biological replicates are shown. (Original uncropped blots can be found in Supporting Information S2: “Original blots for Figure 2A.”) (B) Western blot analysis of HNRNPU expression in SiHa cells treated with glycolytic inhibitors (2‐DG, DCA, or Oxamate). Representative images from three independent biological replicates are shown. (Original uncropped blots can be found in Supporting Information S2: “Original blots for Figure 2B.”) (C) HNRNPU protein stability assay: SiHa cells were pretreated with or without L‐lactate (25 mM) followed by cycloheximide (CHX, 10 µg/mL) for indicated durations. HNRNPU degradation rates were analyzed by Western blot. Representative images from three independent biological replicates are shown. (Original uncropped blots can be found in Supporting Information S2: “Original blots for Figure 2C.”) (D) Western blot of HNRNPU in SiHa cells treated with the proteasome inhibitor MG132. Representative images from three independent biological replicates are shown. (Original uncropped blots can be found in Supporting Information S2: “Original blots for Figure 2D.”) (E) HNRNPU was immunoprecipitated from SiHa cells treated with or without lactate, followed by immunoblotting with an anti‐ubiquitin (Ub) antibody. Representative images from three independent biological replicates are shown. (Original uncropped blots can be found in Supporting Information S2: “Original blots for Figure 2E.”) (F) HNRNPU was immunoprecipitated from cells with or without lactate treatment, and lactylation levels were detected using an anti‐pan‐lactyllysine (Pan‐Kla) antibody. Representative images from three independent biological replicates are shown. (Original uncropped blots can be found in Supporting Information S2: “Original blots for Figure 2F.”) (G) LC–MS/MS analysis identified two candidate lactylation sites in HNRNPU, K181 and K247. Representative MS/MS spectra of the corresponding peptides are shown. The lactylated lysine residues are highlighted in red. (H) Evolutionary conservation analysis of HNRNPU lactylation sites across species. I) Schematic of overlap‐PCR‐generated HNRNPU mutants (K181A/K247A). Mutations were confirmed by Sanger sequencing (lysine‐to‐alanine substitutions, codons GCG/GCA). (J) Immunoblot detection of HNRNPU lactylation in SiHa cells re‐expressing wild‐type (WT) or mutant (K181A/K247A) plasmids. Representative images from three independent biological replicates are shown. (Original uncropped blots can be found in Supporting Information S2: “Original blots for Figure 2J.”) (K) CHX chase assay comparing HNRNPU degradation kinetics in SiHa cells expressing HNRNPU‐WT versus HNRNPU‐K181A. Representative images from three independent biological replicates are shown. (Original uncropped blots can be found in Supporting Information S2: “Original blots for Figure 2K.”) (L) Immunoblot detection of HNRNPU ubiquitination in HeLa cells re‐expressing wild‐type (WT) or mutant (K181A) plasmids. Representative images from three independent biological replicates are shown. (Original uncropped blots can be found in Supporting Information S2: “Original blots for Figure 2L.”).

As HNRNPU regulated the growth of cervical cancer cells both in vivo and in vitro, we hypothesized that the downregulation of HNRNPU might be a potentially feasible strategy to block tumor growth. Thus, clarifying the mechanism of HNRNPU overexpression in cervical cancer was important for subsequent studies. Notably, our systematic screening revealed that HNRNPU protein levels were significantly elevated in cervical cancer tissues despite unchanged mRNA levels (Figure 1A and Figure S1), suggesting post‐translational regulation. Given that lactate accumulation is a hallmark of the cervical cancer microenvironment and emerging evidence indicates that lactate regulates protein expression through lactylation [31, 32], we hypothesized that HNRNPU might be a direct substrate of lactylation in cervical cancer. Consistent with these reports, L‐lactate treatment was found to increase HNRNPU expression in a time‐dependent manner (Figure 2A), whereas inhibition of lactate dehydrogenase activity with 2‐deoxy‐D‐glucose, dichloroacetate or oxamate was observed to reduce HNRNPU levels (Figure 2B). The effect of L‐lactate on HNRNPU half‐life was subsequently examined. Cells were treated with L‐lactate for 36 h prior to cycloheximide (CHX) treatment to block protein synthesis. L‐lactate treatment was found to significantly prolong HNRNPU half‐life compared to controls (Figure 2C), suggesting a role for L‐lactate in regulating HNRNPU protein stability. Protein stability was co‐regulated by multiple post‐translational modifications (PTMs), including ubiquitination and lactylation. Previous studies demonstrated that L‐lactate directly promoted NUSAP1 lactylation to stabilize its expression [33], while also enhancing lactylation through ubiquitination suppression [13]. Accordingly, we investigated the coordinated regulation between ubiquitination and lactylation. MG132 treatment was found to significantly stabilize HNRNPU protein expression (Figure 2D). Moreover, lactate administration was shown to attenuate HNRNPU ubiquitination‐mediated degradation while enhancing lactylation modifications (Figure 2E,F).

To identify lactylation sites, HNRNPU was analyzed by LC‐MS/MS. The MS/MS spectra revealed two lactylated peptides corresponding to K181 and K247, identifying them as lactylation sites in HNRNPU (Figure 2G). Given the high conservation of HNRNPU, it was hypothesized that lactylation‐susceptible regulatory sites would be similarly conserved. Sequence analysis confirmed that K181 and K247 are conserved across species (Figure 2H). To identify the specific residues targeted for lactylation, we mutated each of the two putative lysine residues to alanine (A) (Figure 2I) and subsequently assessed the levels of lactylation. Ectopic expression of K181A was found to significantly inhibit lactylation (Figure 2J). These findings suggest that K181 serves as the major lactylation modification site in HNRNPU. Furthermore, HNRNPU K181A expression was shown to reduce protein half‐life (Figure 2K). To determine the mechanism underlying this instability, we examined the ubiquitination status of the K181A mutant. Consistent with a potential protective role of lactylation, the HNRNPU K181A mutant exhibited increased ubiquitination compared to the wild‐type protein (Figure 2L). Collectively, these results suggest that L‐lactate‐mediated lactylation of HNRNPU at K181 may contribute to its stability by modulating ubiquitination, thereby providing a possible explanation for the reduced stability of the K181A mutant.

### 2.3 HNRNPU K181 Lactylation Promotes Cervical Cancer Cell Proliferation

**FIGURE 3:**
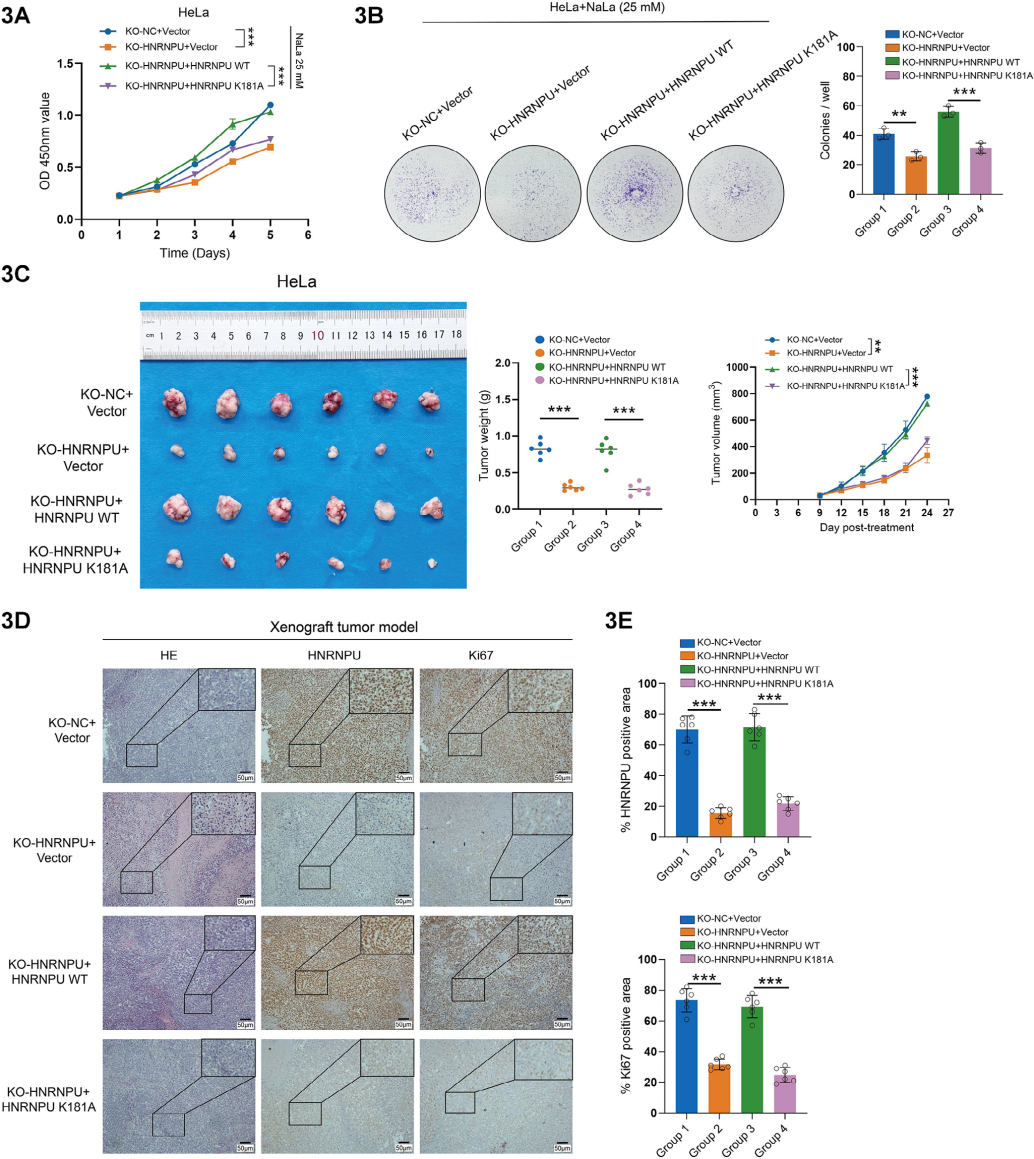
HNRNPU K181 lactylation promotes cervical cancer proliferation in vitro and in vivo. (A) HeLa cells with the indicated treatments were cultured in the presence of sodium L‐lactate (NaLa, 25 mM), and cell proliferation was measured by CCK‐8 assay at the indicated time points. Data are presented as mean ± SD (*n* = 4 per group). Overall growth curves were analyzed using two‐way repeated‐measures ANOVA with group and time as factors. Statistical significance indicated by brackets was determined at the final time point (day 5) using one‐way ANOVA followed by Tukey's multiple comparisons test. ***p < 0.001. (B) Colony formation assay in control or HNRNPU‐knockout HeLa cells reconstituted with empty vector (Vector), wild‐type HNRNPU (HNRNPU WT), or the K181A mutant (HNRNPU K181A) in the presence of sodium L‐lactate (NaLa, 25 mM). The X‐axis labels correspond to the following groups: Group 1, KO‐NC + Vector; Group 2, KO‐HNRNPU + Vector; Group 3, KO‐HNRNPU + HNRNPU WT; Group 4, KO‐HNRNPU + HNRNPU K181A. Data are presented as mean ± SD from three independent biological replicates. Statistical significance was determined by one‐way ANOVA followed by Tukey's multiple comparisons test. **p < 0.01, ***p < 0.001. (C) Xenograft tumor growth of control (KO‐NC) or HNRNPU‐knockout HeLa cells reconstituted with control vector, HNRNPU WT, or HNRNPU K181A under intermittent NaLa treatment. Representative tumor images from each group are shown (*n* = 6 per group). Tumor weights were analyzed using one‐way ANOVA followed by Tukey's multiple comparisons test. The X‐axis labels correspond to the following groups: Group 1, KO‐NC + Vector; Group 2, KO‐HNRNPU + Vector; Group 3, KO‐HNRNPU + HNRNPU WT; Group 4, KO‐HNRNPU + HNRNPU K181A. Tumor volumes were measured every 3 days and are presented as mean ± SD. Overall tumor growth curves were analyzed using two‐way repeated‐measures ANOVA with group and time as factors. Statistical significance indicated by brackets was determined at the final time point (day 24) using one‐way ANOVA followed by Tukey's multiple comparisons test. **p < 0.01, ***p < 0.001. (D, E) Representative images of HE staining and IHC staining of HNRNPU and Ki67 in xenograft tumor sections from each group. Scale bar: 50 µm. Quantification of HNRNPU and Ki67‐positive cells (percentage of positive cells) is shown in the right panel. The X‐axis labels correspond to the following groups: Group 1, KO‐NC + Vector; Group 2, KO‐HNRNPU + Vector; Group 3, KO‐HNRNPU + HNRNPU WT; Group 4, KO‐HNRNPU + HNRNPU K181A. Data are presented as mean ± SD (*n* = 6 mice per group), with each data point representing one mouse. Statistical significance was determined by one‐way ANOVA followed by Tukey's multiple comparisons test. ***p < 0.001.

The functional role of HNRNPU K181 lactylation (HNRNPU K181lac) in cervical cancer cell proliferation was next investigated. To eliminate the influence of endogenous HNRNPU on proliferation, we first generated HNRNPU‐knockout cells. Consistent with the above findings, loss of HNRNPU significantly impaired cellular proliferation. In sodium L‐lactate (NaLa)‐treated cells, re‐expression of wild‐type HNRNPU, but not the K181A mutant, significantly rescued the proliferation defect caused by HNRNPU deficiency (Figure 3A,B). To further evaluate the oncogenic role of HNRNPU K181lac in vivo, a high‐lactate tumor microenvironment was established by intraperitoneal NaLa administration. Consistent with the in vitro results, the impaired tumor growth observed in HNRNPU‐knockout xenografts was specifically rescued by wild‐type HNRNPU, but not by the K181A mutant (Figure 3C,D). These findings demonstrate that HNRNPU K181 lactylation promotes cervical cancer proliferation both in vitro and in vivo.

### 2.4 NAA50‐Mediated Acetylation Competitively Inhibits HNRNPU K181 Lactylation

**FIGURE 4:**
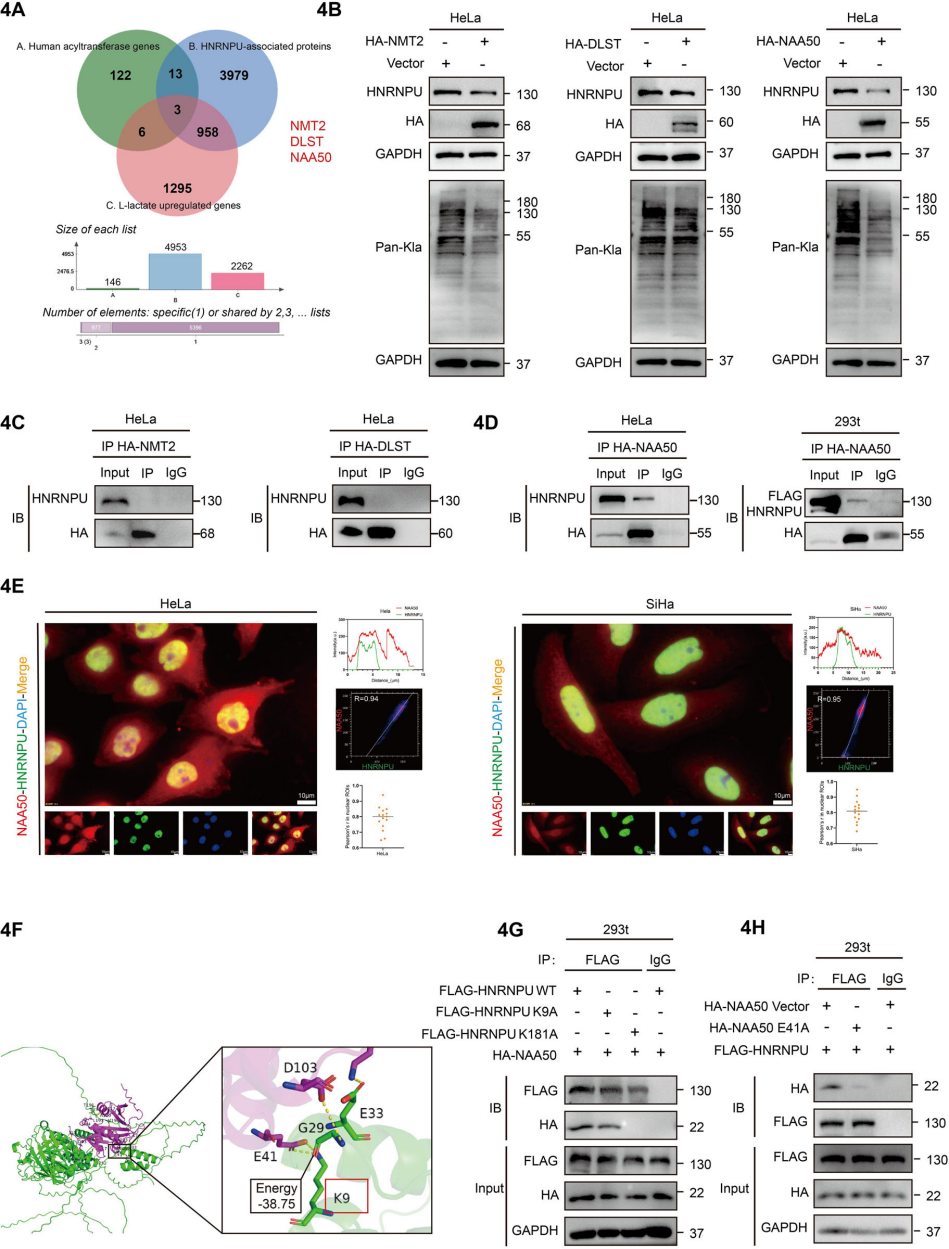
NAA50 mediates HNRNPU lactylation through direct interaction. (A) Venn diagram showing the intersection of HNRNPU‐associated proteins (4953 candidates), L‐lactate‐upregulated transcripts (2262 candidates), and known acyltransferases (144 candidates), identifying three candidate regulators (NMT2, DLST, NAA50). (B) Western blot analysis of HNRNPU protein levels and pan‐lactylation in HeLa cells overexpressing NMT2, DLST, or NAA50 (with Vector control). Representative images from three independent biological replicates are shown. (Original uncropped blots can be found in Supporting Information S2: “Original blots for Figure 4B.”) (C) Co‐IP assays testing interactions between HNRNPU and candidate acyltransferases. Lysates from HeLa cells transfected with HA‐tagged NMT2/DLST were immunoprecipitated with anti‐HA antibody and probed with anti‐HNRNPU. Representative images from three independent biological replicates are shown. (Original uncropped blots can be found in Supporting Information S2: “Original blots for Figure 4C.”) (D) Endogenous and exogenous Co‐IP validation of NAA50‐HNRNPU interaction. Left: Endogenous proteins immunoprecipitated from HeLa cells. Right: Exogenous interaction in 293T cells co‐transfected with FLAG‐HNRNPU and HA‐NAA50. Representative images from three independent biological replicates are shown. (Original uncropped blots can be found in Supporting Information S2: “Original blots for Figure 4D.”) (E) Colocalization of NAA50 and HNRNPU in HeLa and SiHa cells. Representative immunofluorescence images of NAA50 (red), HNRNPU (green), and DAPI (blue), together with representative line‐scan profiles and 2D intensity histograms. Pearson's correlation coefficients were quantified in nuclear ROIs from individual cells (*n* = 15 cells per group). Scale bar: 10 µm. (F) Molecular docking model of NAA50‐HNRNPU interaction. Left: Predicted binding interface showing NAA50 E41 (magenta) and HNRNPU K9 (cyan). Right: Binding energy heatmap (kcal/mol). (G) Co‐IP analysis of HA‐NAA50 binding to FLAG‐tagged HNRNPU mutants (K9A, K181A). Lysates from 293T cells co‐transfected with indicated plasmids were immunoprecipitated with anti‐FLAG and probed with anti‐HA. Representative images from three independent biological replicates are shown. (Original uncropped blots can be found in Supporting Information S2: “Original blots for Figure 4G.”) (H) Co‐IP assay testing interaction between FLAG‐HNRNPU and HA‐tagged NAA50 E41A mutant. Representative images from three independent biological replicates are shown. (Original uncropped blots can be found in Supporting Information S2: “Original blots for Figure 4H.”).

**FIGURE 5:**
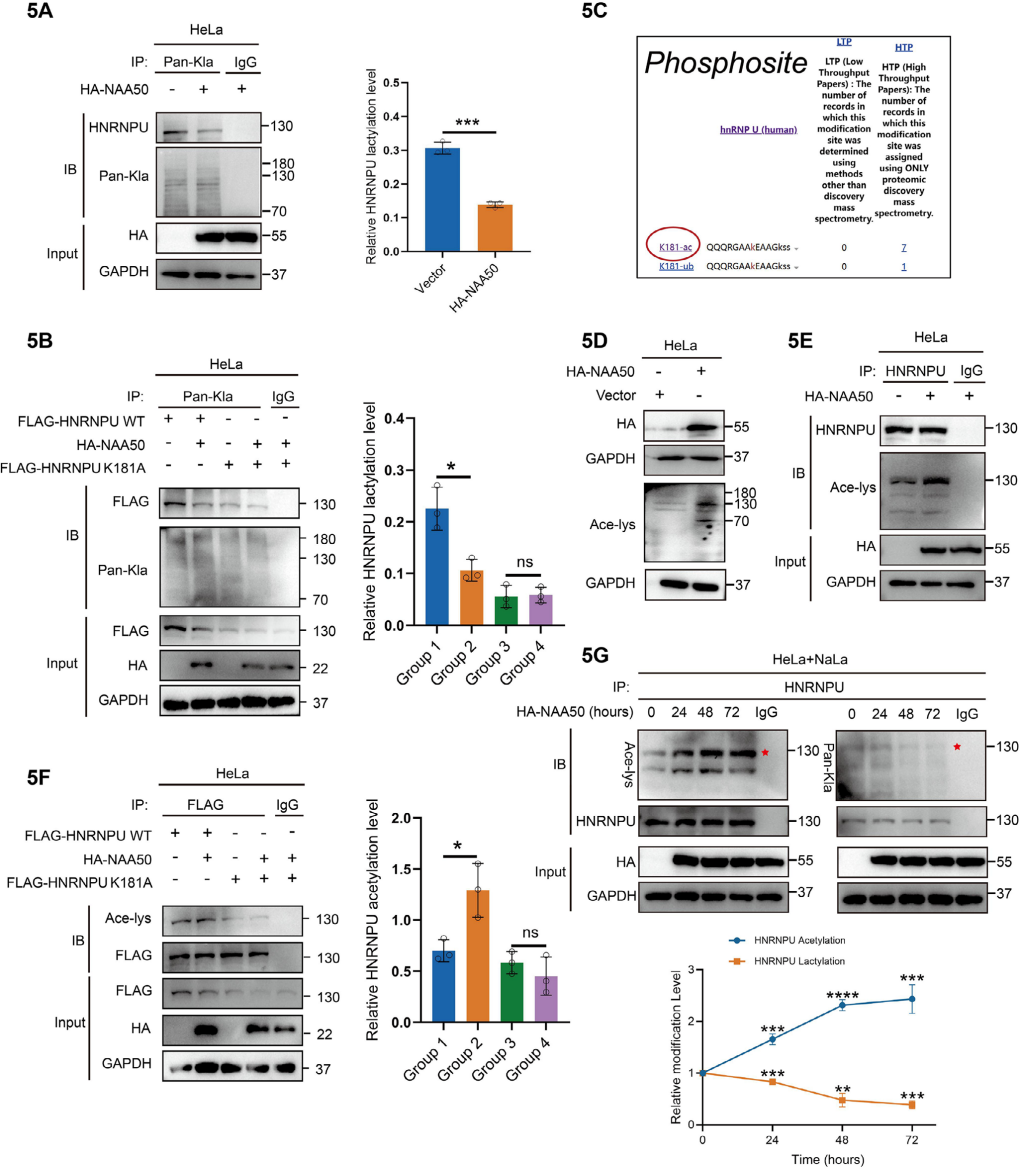
NAA50 competitively regulates HNRNPU acetylation and lactylation at K181. (A) Western blot analysis of pan‐lactylated proteins immunoprecipitated from HeLa cells overexpressing NAA50 or vector and probed with anti‐HNRNPU antibody. A representative blot is shown. Quantification of HNRNPU lactylation levels was performed from three independent biological replicates. Data are presented as mean ± SD. Statistical significance was determined by two‐tailed unpaired Student's t‐test. ***p < 0.001. (Original uncropped blots can be found in Supporting Information S2: “Original blots for Figure 5A.”) (B) Lactylation levels of wild‐type (WT) and K181A mutant HNRNPU in the presence or absence of NAA50 overexpression. Representative images from three independent biological replicates are shown. The X‐axis labels correspond to the following groups: Group 1, FLAG‐HNRNPU WT; Group 2, FLAG‐HNRNPU WT + HA‐NAA50; Group 3, FLAG‐HNRNPU K181A; Group 4, FLAG‐HNRNPU K181A + HA‐NAA50. Data are presented as mean ± SD (*n* = 3 per group). Statistical significance was determined using one‐way ANOVA followed by Tukey's multiple comparisons test. *p < 0.05; ns, not significant. (Original uncropped blots can be found in Supporting Information S2: “Original blots for Figure 5B.”) (C) Screenshot from PhosphoSitePlus database showing HNRNPU‐K181 as a documented acetylation site. (D) Global acetylation changes in NAA50‐overexpressing cells detected by anti‐acetyl‐lysine Western blot. Representative images from three independent biological replicates are shown. (Original uncropped blots can be found in Supporting Information S2: “Original blots for Figure 5D.”) (E) HNRNPU acetylation analysis via immunoprecipitation (IP) with anti‐HNRNPU followed by anti‐acetyl‐lysine immunoblotting in NAA50‐overexpressing cells. Representative images from three independent biological replicates are shown. (Original uncropped blots can be found in Supporting Information S2: “Original blots for Figure 5E.”) (F) Acetylation levels of wild‐type (WT) and K181A mutant HNRNPU in the presence or absence of NAA50 overexpression. Representative images from three independent biological replicates are shown. The X‐axis labels correspond to the following groups: Group 1, FLAG‐HNRNPU WT; Group 2, FLAG‐HNRNPU WT + HA‐NAA50; Group 3, FLAG‐HNRNPU K181A; Group 4, FLAG‐HNRNPU K181A + HA‐NAA50. Data are presented as mean ± SD (*n* = 3 per group). Statistical significance was determined using one‐way ANOVA followed by Tukey's multiple comparisons test. *p < 0.05; ns, not significant. (Original uncropped blots can be found in Supporting Information S2: “Original blots for Figure 5F.”) (G) Time‐course analysis of HNRNPU acetylation and lactylation in lactate‐treated (25 mM) cells following NAA50 overexpression. Representative immunoblot images from three independent biological replicates are shown. The asterisk indicates the band corresponding to acetylated and lactylated HNRNPU. Quantification of relative acetylation and lactylation levels, normalized to total HNRNPU, is shown in the bottom panels. Data are presented as mean ± SD from three independent biological replicates. Statistical significance for acetylation and lactylation was analyzed separately using one‐way ANOVA followed by Dunnett's multiple comparisons test, with each time point compared with 0 h. **p < 0.01, ***p < 0.001, ****p < 0.0001. (Original uncropped blots can be found in Supporting Information S2: “Original blots for Figure 5G.”).

To investigate the regulatory mechanism underlying HNRNPU lactylation, we sought to identify potential upstream enzymes responsible for this modification. Since lysine lactylation is typically catalyzed by acyltransferases [34], we first performed immunoprecipitation followed by liquid chromatography–tandem mass spectrometry (IP–LC–MS/MS) to identify proteins associated with HNRNPU, resulting in the identification of 4,953 HNRNPU‐associated proteins. To further prioritize candidate regulators involved in HNRNPU lactylation, these proteins were integrated with two additional datasets. First, transcripts significantly upregulated upon L‐lactate treatment in HeLa cells were identified based on our RNA‐seq analysis. Second, a curated list of human acyltransferase‐related genes was obtained from the UniProt database (Homo sapiens) based on functional annotations. Intersection of these three datasets identified three overlapping genes—N‐myristoyltransferase 2 (NMT2), dihydrolipoamide S‐succinyltransferase (DLST), and N‐alpha‐acetyltransferase 50 (NAA50), suggesting that these genes may serve as potential upstream regulators of HNRNPU lactylation (Figure 4A). Notably, NAA50 overexpression significantly reduced both HNRNPU expression and pan‐lactylation levels (Figure 4B). Co‐immunoprecipitation (Co‐IP) assays were subsequently performed to validate these interactions. While no interaction was detected between HNRNPU and either NMT2 or DLST (Figure 4C), both endogenous and exogenous NAA50 demonstrated robust binding to HNRNPU (Figure 4D). Immunofluorescence confirmed NAA50‐HNRNPU colocalization in HeLa and SiHa cells (Figure 4E). To characterize the molecular basis of this interaction, we performed molecular docking analysis using AlphaFold3. The structural prediction identified a potential interaction interface wherein NAA50 residue E41 forms a stable electrostatic interaction with HNRNPU residue K9, with a binding energy of −38.75 kcal/mol (Figure 4F). Notably, no direct interaction was predicted between NAA50 E41 and HNRNPU K181. AlphaFold3 analysis further supported this interaction model, showing a significant iPTM score of 0.49 (threshold >0.3) (Figure 2A). To experimentally validate the predicted interface, we generated FLAG‐tagged HNRNPU mutants (K9A and K181A) and assessed their binding to HA‐NAA50 by Co‐IP. Consistent with the docking prediction, disrupting the predicted direct contact point (HNRNPU K9A) significantly weakened the interaction with NAA50 (Figure 4G). Notably, the K181A mutation—despite not being identified as a direct binding partner in the docking analysis—also completely disrupted NAA50 binding (Figure 4G). This finding indicates that while K9 serves as a predicted direct contact residue, K181 is also critically required for the HNRNPU‐NAA50 interaction, suggesting that the binding interface involves additional determinants beyond the predicted E41‐K9 contact. Conversely, we generated an HA‐tagged NAA50 E41A mutant, in which glutamic acid 41 was replaced with alanine (E41A), and assessed its binding to wild‐type HNRNPU. This mutation completely abolished the interaction with HNRNPU (Figure 4H), confirming E41 as a critical interaction residue on NAA50. These results demonstrate that the HNRNPU‐NAA50 interaction requires both the predicted contact residue K9 and an additional essential residue K181, with NAA50 E41 serving as the key complementary binding determinant. Given the essential role of K181 in this interaction, we next investigated whether NAA50 regulates HNRNPU post‐translational modifications by targeting this residue. First, we performed immunoprecipitation (IP) to enrich pan‐lactylated proteins, followed by Western blot analysis. The results showed that NAA50 overexpression significantly reduced HNRNPU lactylation (Figure 5A). To define the role of K181 in NAA50‐mediated regulation, we compared the lactylation status of wild‐type (WT) and K181A mutant HNRNPU in the presence or absence of NAA50 co‐expression. The results showed that NAA50 overexpression inhibited the lactylation of WT HNRNPU. In contrast, lactylation of the K181A mutant is already lower than that of the WT and is not further reduced upon NAA50 co‐expression (Figure 5B). The observations suggest that the effect of NAA50 on HNRNPU lactylation depends on the presence of the K181 residue. This observation appeared contradictory to our initial hypothesis that NAA50, as an acyltransferase, might promote HNRNPU lactylation. Previous studies have reported that both acetylation and lactylation occur on lysine residues of proteins. We therefore questioned whether HNRNPU K181 could undergo both modifications and whether NAA50 might coordinately regulate these two PTMs, potentially through competitive inhibition. To address this, we first queried the PhosphoSitePlus database, which confirmed that HNRNPU‐K181 is a known acetylation site (Figure 5C). Subsequent experiments revealed that NAA50 overexpression increased global acetylation levels (Figure 5D) as well as HNRNPU acetylation (Figure 5E). Notably, the HNRNPU K181A mutant eliminates the NAA50‐induced increase in acetylation (Figure 5F), indicating that NAA50‐mediated acetylation depends on the presence of the K181 residue. These results are consistent with a model in which acetylation at K181 may influence lactylation at the same site. To dynamically monitor the temporal changes in these modifications, we exogenously added lactate and tracked HNRNPU acetylation and lactylation over time. Using HNRNPU antibody‐based enrichment, we observed that NAA50 treatment led to a progressive increase in HNRNPU acetylation accompanied by a gradual decrease in lactylation. Quantitative analysis demonstrated that acetylation levels increased significantly over time, whereas lactylation levels showed a corresponding decrease, revealing an inverse temporal pattern between these modifications (Figure 5G). Intriguingly, NAA50 and HNRNPU mRNA levels were positively correlated in cervical tumors (Figure S2B), suggesting that they may be co‐expressed at the transcriptional level. However, the underlying basis of this correlation remains to be determined. This observation is consistent with the possibility that the inhibitory effect of NAA50 on HNRNPU function, as defined in our study, is mediated primarily through post‐translational modification (acetylation at K181), rather than through transcriptional suppression. In summary, these data demonstrate that NAA50 targets HNRNPU at K181 to promote acetylation, which competitively suppresses lactylation levels.

Furthermore, recent studies have identified AARS1 as a protein with lactyltransferase activity. To investigate whether AARS1 is involved in HNRNPU lactylation, we performed shRNA‐mediated knockdown of AARS1 in HeLa cells. As shown in Figure S2C, immunoprecipitation using a pan‐Kla antibody followed by immunoblotting for HNRNPU revealed that AARS1 knockdown was associated with a reduction in HNRNPU lactylation compared to control cells, suggesting that HNRNPU may be a potential substrate of AARS1. To further explore the relationship between AARS1 and NAA50, which we previously identified as a regulator of HNRNPU lactylation, we performed combined knockdown and overexpression experiments. As shown in Figure S2D, AARS1 knockdown alone reduced HNRNPU lactylation, while overexpression of HA‐NAA50 in AARS1‐depleted cells led to a further decrease in lactylation levels. These findings suggest that AARS1 and NAA50 may both contribute to the regulation of HNRNPU lactylation, although the precise relationship between these factors remains to be clarified.

### 2.5 HNRNPU K181 Lactylation Activates the Serine Metabolism Pathway

**FIGURE 6:**
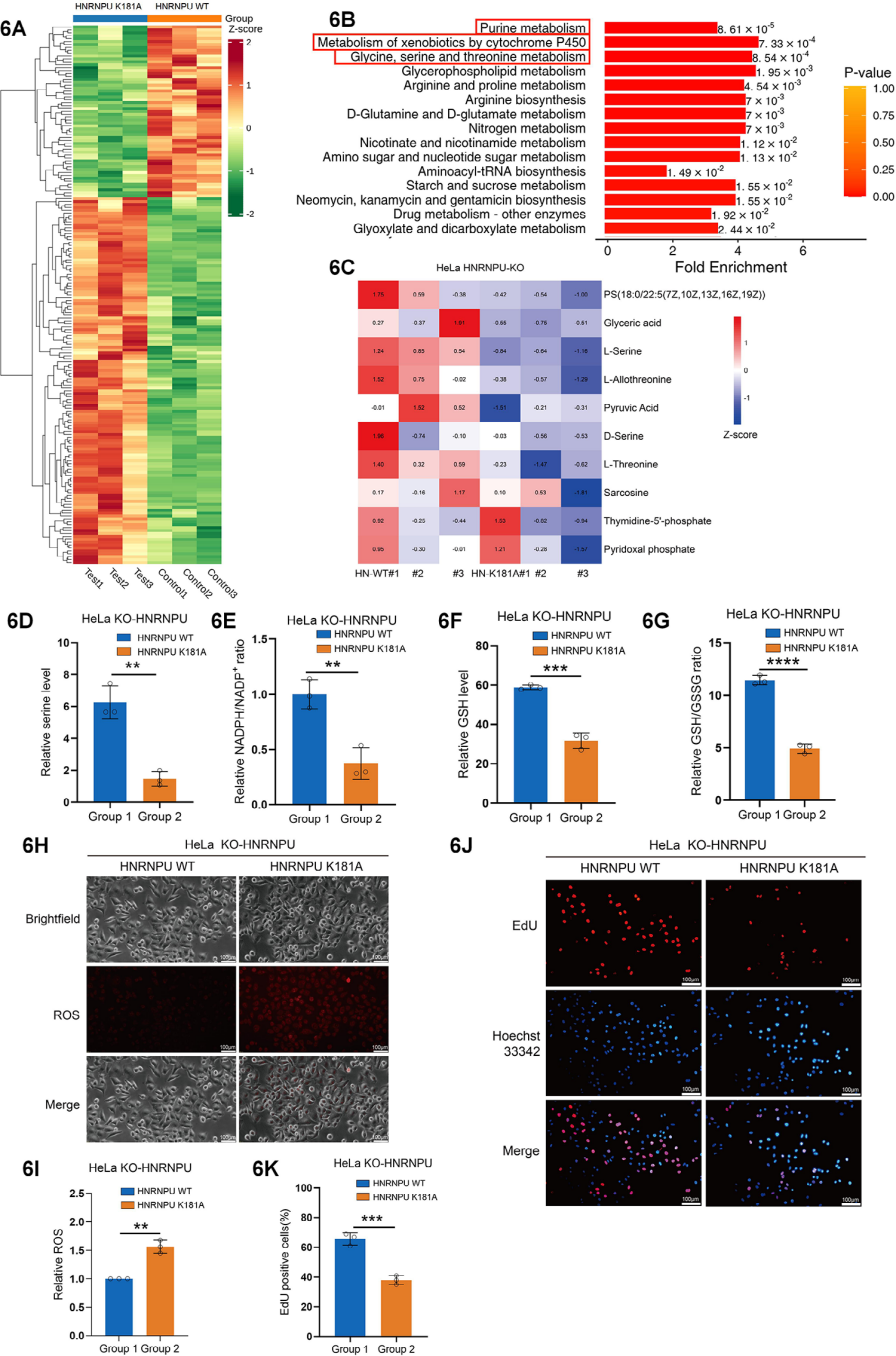
HNRNPU K181 lactylation activates the serine metabolism pathway. (A) Hierarchical clustering heatmap of differentially abundant metabolites between HNRNPU K181A mutant cells (Test) and wild‐type controls (Control). Rows represent individual metabolites; columns represent individual samples. Red indicates high relative abundance; green indicates low relative abundance. B) KEGG pathway enrichment analysis of differentially abundant metabolites. The top 15 enriched pathways are shown. Glycine, serine, and threonine metabolism was ranked third (*p* = 8.54 × 10^−^^4^), with purine metabolism (*p* = 8.61×10^−^^5^) and xenobiotic metabolism (*p* = 7.33 × 10^−^^4^) ranking first and second, respectively. (C) Targeted quantification of key serine pathway metabolites (L‐serine, glyceric acid, and pyruvic acid) in HNRNPU K181A mutant cells versus controls. Data are presented as mean ± SEM from three independent biological replicates. (D–G) Functional validation of serine metabolism in HeLa KO‐HNRNPU cells. The X‐axis labels correspond to the following groups: Group 1, HNRNPU WT; Group 2, HNRNPU K181A. (D) Intracellular serine levels. (E) NADPH/NADP^+^ ratio. (F) Total GSH content. (G) GSH/GSSG ratio. Data are presented as mean ± SD from three independent biological replicates. Statistical significance was determined by two‐tailed unpaired t‐test. **p < 0.01, ***P <0.001, ****p <0.0001. (H, I) ROS accumulation measured by DCFH‐DA fluorescence in HeLa cells expressing HNRNPU K181A versus Vector. Scale bar: 50 µm. The X‐axis labels correspond to the following groups: Group 1, HNRNPU WT; Group 2, HNRNPU K181A. Data are presented as mean ± SD from three independent biological replicates. Statistical significance was determined by unpaired two‐tailed Student's t‐test. **p < 0.01. (J, K) DNA synthesis assessed by EdU incorporation in HeLa cells Scale bar: 100 µm. The X‐axis labels correspond to the following groups: Group 1, HNRNPU WT; Group 2, HNRNPU K181A. Data are presented as mean ± SD from three independent biological replicates. Statistical significance was determined by unpaired two‐tailed Student's *t*‐test. ***p <0.001.

To systematically investigate the downstream metabolic pathways regulated by HNRNPU K181 lactylation, we performed unbiased global metabolomic profiling comparing HNRNPU K181A mutant cells with wild‐type controls. OPLS‐DA analysis revealed a clear separation between K181A mutant and control cells (Figure S3A), with robust model parameters (R^2^X = 0.538, R^2^Y = 1.00, Q^2^ = 0.708; permutation test *p* < 0.005) (Figure S3B), indicating that loss of K181 lactylation induces substantial and reliable metabolic reprogramming. Hierarchical clustering analysis further confirmed distinct metabolic patterns between K181A and control groups, with good within‐group reproducibility (Figure 6A). KEGG pathway enrichment analysis of all differentially abundant metabolites identified multiple significantly altered pathways (Figure 6B). The top three ranked pathways were purine metabolism (*p* = 8.61 × 10^−^^5^), metabolism of xenobiotics by cytochrome P450 (*p* = 7.33 × 10^−^^4^), and glycine, serine, and threonine metabolism (*p* = 8.54 × 10^−^^4^). Closer examination of these three pathways revealed that although statistically significant, the xenobiotic metabolism pathway is primarily involved in detoxification of exogenous compounds and was not prioritized for further investigation in the current work; therefore, it was not pursued further. Notably, purine metabolism and serine metabolism are intrinsically linked—serine provides essential one‐carbon units for purine synthesis via one‐carbon metabolism, positioning serine metabolism upstream of purine metabolism. When comparing the enrichment magnitudes of these two pathways, we found that despite its slightly higher *p*‐value, serine metabolism exhibited a higher fold enrichment than purine metabolism, suggesting a more pronounced magnitude of change in its metabolite levels. Given that serine lies upstream of purine metabolism, alterations in serine flux may secondarily impact purine metabolism, making serine a potentially more direct target of HNRNPU‐mediated regulation. Furthermore, the serine synthesis pathway has well‐defined rate‐limiting enzymes, providing an ideal entry point for subsequent investigation of how HNRNPU regulates metabolic gene expression through post‐transcriptional mechanisms. Importantly, serine metabolism is intimately linked to redox homeostasis through glutathione biosynthesis and supports nucleotide synthesis via one‐carbon metabolism—processes central to cancer cell proliferation. Based on this integrated analysis, we focused our subsequent investigation on glycine, serine and threonine metabolism. Targeted metabolite quantification confirmed that key metabolites within this pathway, including L‐serine, glyceric acid, and pyruvic acid, were significantly reduced in K181A mutant cells (Figure 6C), consistent with the pathway enrichment analysis. Specifically, quantitative analysis confirmed significant decreases in the abundance of these key metabolites (Figure S3C). Serine is synthesized from the glycolytic intermediate 3‐phosphoglycerate by the rate‐limiting enzyme phosphoglycerate dehydrogenase (PHGDH) and serves multiple metabolic functions. As both a proteinogenic amino acid and a glycine precursor, serine directly supports nucleotide biosynthesis and DNA replication. Additionally, serine contributes to glutathione biosynthesis, maintaining cellular redox homeostasis. We therefore further investigated the functional impact of HNRNPU K181 lactylation on serine metabolism. In both HeLa KO‐HNRNPU and C33A cells, HNRNPU K181A expression significantly reduced intracellular serine levels, the NADPH/NADP^+^ ratio, and glutathione (GSH/GSSG) redox status compared to controls (Figure 6D–G and Figure S3D–G). Consistent with these findings, ROS accumulation was significantly increased in K181A‐expressing cells (Figure 6H,I), and DNA synthesis was markedly suppressed (Figure 6J,K). Together, these results demonstrate that HNRNPU K181 lactylation functionally activates the serine biosynthesis pathway to support redox homeostasis and DNA replication in cervical cancer cells.

### 2.6 HNRNPU K181 Maintains the Exon 1‐Containing PHGDH Transcript and mRNA Stability Through Direct Transcript Binding

**FIGURE 7:**
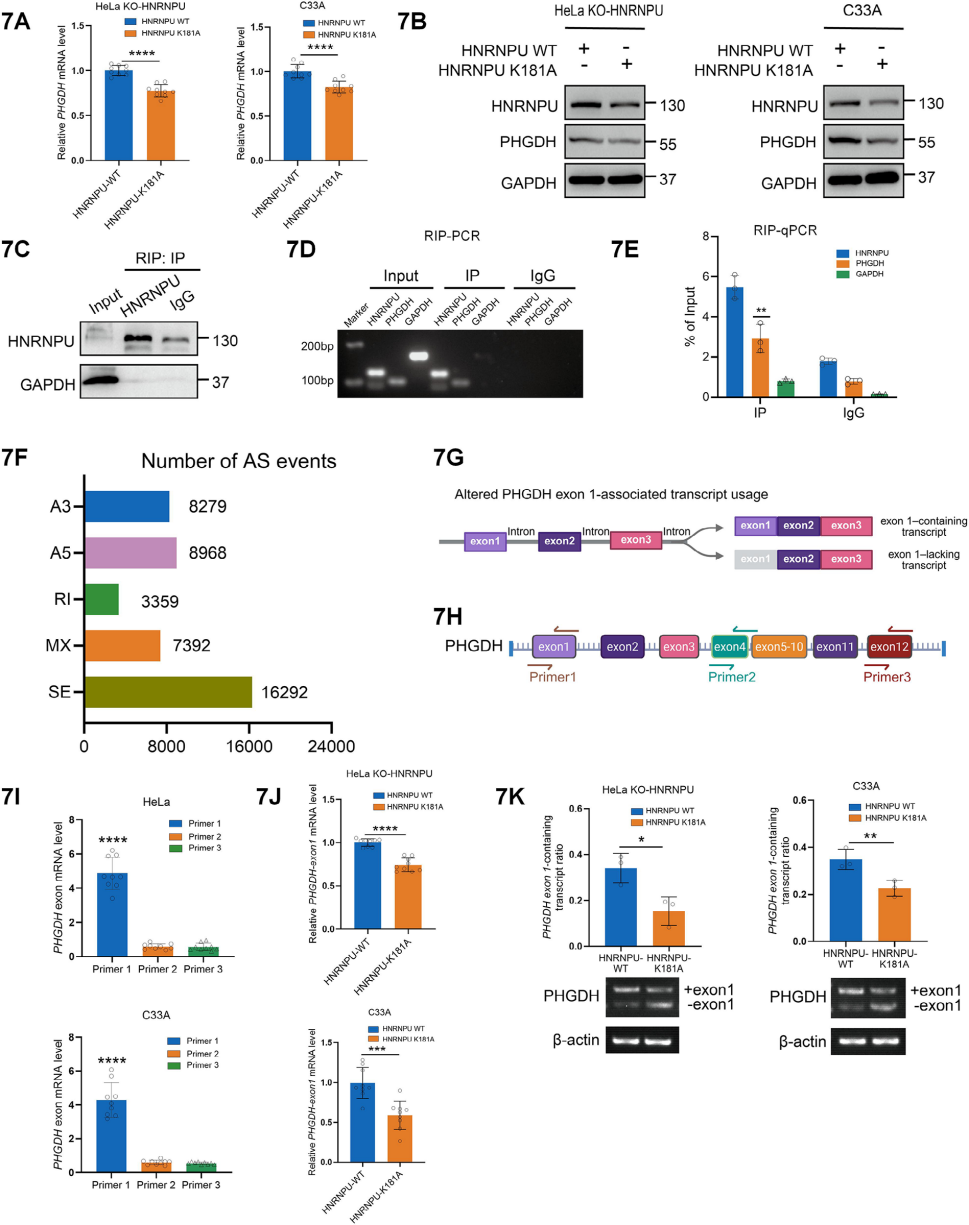
HNRNPU K181 lactylation regulates PHGDH expression through altered exon 1‐associated transcript usage. (A, B) PHGDH expression analysis in HNRNPU K181A‐expressing cells: (A) qRT‐PCR of PHGDH mRNA levels. Data are presented as mean ± SD from three independent biological replicates, each performed with three technical replicates. Statistical significance was determined by unpaired two‐tailed Student's t‐test. ***p < 0.001. (B) Western blot of PHGDH protein. (Original uncropped blots can be found in Supporting Information S2: “Original blots for Figure 7B.”) (C) Validation of RNA immunoprecipitation (RIP) efficiency. Western blot showing enrichment of HNRNPU protein in RIP complexes compared with IgG control. Representative images from three independent biological replicates are shown. (Original uncropped blots can be found in Supporting Information S2: “Original blots for Figure 7C.”) (D) RIP‐PCR analysis of PHGDH mRNA enrichment. Endpoint PCR was performed using RNA isolated from the RIP complexes shown in (C). Representative gel images from three independent biological replicates are shown. (E) RIP‐qPCR quantification of PHGDH mRNA enrichment in HNRNPU immunoprecipitates relative to IgG control. Data are presented as mean ± SD from three independent biological replicates; technical triplicates were averaged for each biological replicate prior to statistical analysis. Statistical significance was assessed using a two‐tailed paired t‐test. **p < 0.01. (F) Quantification of the different AS events affected by HNRNPU K181. (G) Schematic of PHGDH alternative splicing isoforms. Schematic representation of alternative splicing patterns in PHGDH. (H) Design of specific primers labeled as primer 1 to primer 3. (I) RIP assays to detect the specific interaction sites between HNRNPU and PHGDH. Data are presented as mean ± SD from three independent experiments. ****p < 0.0001. (J) RT qPCR assay showing PHGDH exon 1 expression in HeLa and C33A cells. Data are presented as mean ± SD from three independent biological replicates, each performed with three technical replicates. Statistical significance was determined by unpaired two‐tailed Student's *t*‐test. ****p < 0.0001. (K) RT‐PCR assay showing the ratio of the exon 1‐containing PHGDH transcript in HeLa and C33A cells with HNRNPU WT or HNRNPU K181A. Representative gel images from three independent biological replicates are shown. Quantification of ratio values is shown in the top panel. Data are presented as mean ± SD from three independent experiments. Statistical significance was determined by unpaired two‐tailed Student's t‐test. *p < 0.05, **p < 0.01.

Given the observed suppression of serine pathway activity by HNRNPU K181A, we further examined the expression of three key enzymes in the serine synthesis pathway—PHGDH, PSAT1, and PSPH. The results showed that HNRNPU K181A expression specifically downregulated the mRNA level of PHGDH, whereas the expression of PSAT1 and PSPH remained unaffected (Figure 7A and Figure S4A,B). Similarly, HNRNPU K181A significantly suppressed the protein expression of PHGDH (Figure 7B). These findings indicate that HNRNPU lactylation specifically regulates the rate‐limiting enzyme PHGDH in the serine synthesis pathway. Analysis of the TCGA cervical cancer (CESC) cohort identified a significant positive correlation between HNRNPU and PHGDH expression (*R* = 0.20, *p* = 0.0004). In stark contrast, no significant correlation was observed in a large compendium of normal tissues (*R* = 0.059, *p* = 0.12), revealing a tumor‐specific co‐expression pattern (Figure S4C). Elevated PHGDH expression levels were positively correlated with poor prognosis and reduced survival in cancer patients (Figure S4D). As HNRNPU is an RNA‐binding protein known to regulate alternative splicing events, it was hypothesized that it may modulate PHGDH expression via transcript binding. RNA immunoprecipitation (RIP) assays were performed to validate this interaction. Successful enrichment of HNRNPU was confirmed by immunoblotting (Figure 7C). PHGDH mRNA was detected in the immunoprecipitated complexes by both endpoint RIP‐PCR and RIP‐qPCR (Figure 7D,E), indicating that HNRNPU directly associates with PHGDH mRNA.

Given that HNRNPU K181A markedly affects PHGDH expression and that HNRNPU functions as an RNA‐binding protein involved in alternative splicing regulation, we performed third‐generation sequencing to investigate whether PHGDH transcript structure is altered, while simultaneously assessing global changes in transcriptome‐wide splicing patterns. Widespread RNA splicing alterations were observed in the HNRNPU K181A group, with exon skipping representing the predominant event at the transcriptome‐wide level (Figure 7F). A comprehensive list of the identified splicing events is provided in Table S1. In addition, pathway enrichment analysis of differential splicing genes derived from the most frequent SE (skipped exon) events revealed significant enrichment in metabolic pathways, including carbon metabolism and the tricarboxylic acid (TCA) cycle, as well as RNA processing‐related pathways such as the spliceosome and RNA polymerase (Figure S5A). These results indicate that HNRNPU K181A induces functionally directed, rather than random, transcriptome‐wide splicing alterations. On this basis, we further focused on PHGDH to investigate the transcript structural basis underlying its expression changes mediated by HNRNPU K181A. Visualization of read coverage and splice‐junction patterns revealed that, in the K181A group, the 5′ region of PHGDH—particularly the first exon‐associated region—displayed distinct read distribution and splice‐junction usage compared with WT cells. Notably, splice‐junction signals directly connecting the exon 1‐adjacent region to the downstream exon (exon 2) were observed in the K181A group but were less apparent in WT cells, indicating altered first exon‐associated transcript usage of PHGDH in the HNRNPU K181A condition (Figure S5B). To further clarify the splice‐junction pattern of PHGDH, we generated a sashimi plot based on the sequencing data, focusing on the region spanning PHGDH exon 1 and exon 2. Compared with HNRNPU WT cells, HNRNPU K181A cells showed a marked reduction in canonical exon 1–exon 2 junction reads, whereas reads supporting the exon 1 coding‐region‐to‐exon 2 alternative junction were increased (Figure S5C). These results suggest that HNRNPU K181A is associated with increased usage of the exon 1 coding‐region‐to‐exon 2 alternative junction. Consistently, PSI analysis of the PHGDH exon 1–associated event also showed a marked reduction in the HNRNPU K181A group compared with WT, with the corresponding PSI values and statistical results provided in Table S1. Based on these observations, we generated a schematic model summarizing the first exon‐associated transcript changes of PHGDH, illustrating altered 5′‐end transcript usage under the HNRNPU K181A condition (Figure 7G). To further define the binding region of HNRNPU, specific primers (primers 1–3) were designed (Figure 7H). RNA immunoprecipitation (RIP) assays demonstrated preferential enrichment of HNRNPU at the exon 1 region of PHGDH RNA (Figure 7I). We then evaluated the effect of HNRNPU K181A on exon 1‐containing PHGDH transcripts in HeLa and C33A cells. The results showed that HNRNPU K181A reduced the abundance of exon 1‐containing PHGDH transcripts (Figure 7J) and decreased the corresponding exon 1–containing transcript ratio value (Figure 7K).

To determine whether the effect of K181A on PHGDH is due to the site‐specific mutation or reflects a broader disruption of HNRNPU‐dependent RNA regulation, we examined another previously reported HNRNPU‐regulated transcript, CCL2 [35]. RT‐qPCR analysis showed that the K181A mutation had no significant effect on the overall mRNA expression of CCL2 (Figure S5D). In contrast, under the same experimental conditions, PHGDH consistently exhibited reduced exon 1‐containing transcript abundance along with altered exon 1‐associated RNA processing, suggesting that the K181A mutation exerts a transcript‐selective effect. To further evaluate this at a broader level, we performed an orthogonal comparative analysis using a published HNRNPU dataset generated in HeLa cells, which includes both HNRNPU cyto‐CLIP binding data and HNRNPU knockdown RNA‐seq data (GSE138726) [36]. Genes that were both bound by HNRNPU and altered upon its knockdown were defined as HNRNPU direct‐functional target candidates (Table S2). We then compared this gene set with the significant DAS/DTU genes identified in our HNRNPU K181A sequencing dataset. Notably, only 26.8% of these direct‐functional target candidates overlapped with significant DAS/DTU events, whereas 73.2% showed no significant DAS/DTU changes under the K181A condition (Figure S5E). These results suggest that the K181A mutation does not cause a global collapse of HNRNPU‐dependent RNA regulatory activity, but instead exerts selective effects on a subset of downstream transcripts. Based on these findings, the present study further focuses on the transcript‐level regulatory mechanism of PHGDH mediated by HNRNPU K181A.

**FIGURE 8:**
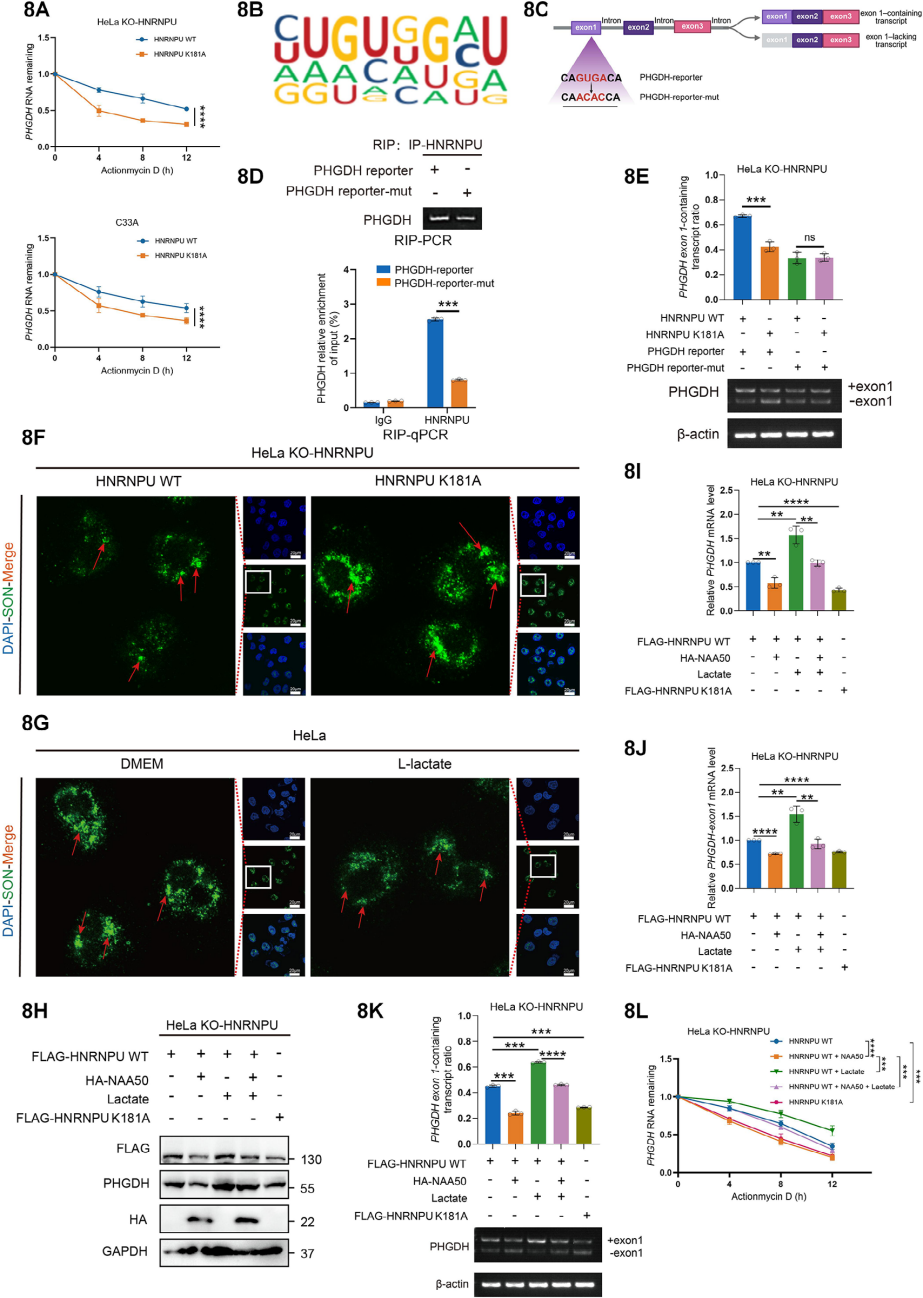
HNRNPU K181 maintains the exon 1‐containing PHGDH transcript through sequence‐specific binding and nuclear speckle organization. (A) Actinomycin D chase assay showing PHGDH mRNA decay kinetics in HeLa and C33A cells reconstituted with HNRNPU WT or HNRNPU K181A following treatment with actinomycin D (10 µg/mL) for 0, 4, 8, and 12 h, reflecting changes in PHGDH mRNA stability. PHGDH mRNA levels were measured by qRT‐PCR and normalized to the 0 h group. Data are presented as mean ± SD (*n* = 9). Decay curves were analyzed using two‐way repeated‐measures ANOVA, followed by Sidak's multiple‐comparisons test. Only selected pairwise comparisons at the 12 h time point are shown for clarity. ****p < 0.0001. (B) Sequence alignment of predicted HNRNPU binding motif. (C) The schematic of PHGDH‐reporter and PHGDH‐reporter‐mut. (D) RIP‐PCR and RIP‐qPCR validation of the interaction between HNRNPU and PHGDH reporter transcripts containing the wild‐type or mutant binding site. Representative RIP‐PCR results are shown in the upper panel, and RIP‐qPCR quantification of PHGDH enrichment in HNRNPU immunoprecipitates is shown in the lower panel. Data are presented as mean ± SD from three independent biological replicates. Statistical significance was determined using a two‐tailed unpaired *t*‐test. ***p < 0.001. (E) Analysis of exon 1–associated PHGDH transcript usage using PHGDH reporter or PHGDH reporter‐mut constructs in HeLa cells. Representative RT‐PCR results are shown in the lower panel, indicating exon 1–containing (+exon1) and exon 1–lacking (−exon1) transcripts. Quantification of the exon 1–containing transcript ratio is shown in the upper panel. Data are presented as mean ± SD from three independent biological replicates (*n* = 3). Statistical significance was determined using one‐way ANOVA followed by Dunnett's multiple comparisons test. F) Representative immunofluorescence images of SON staining (a nuclear speckle marker) in HeLa HNRNPU KO cells expressing HNRNPU WT or the HNRNPU K181A mutant. Compared with HNRNPU WT, expression of the K181A mutant led to larger and more aggregated SON‐positive nuclear speckles. Experiments were performed three times independently. Scale bar: 20 µm. G) Representative immunofluorescence images of SON staining in HeLa cells treated with or without L‐lactate (25 mM, 24 h). Compared with the DMEM control group, L‐lactate treatment led to more dispersed and finer SON‐positive nuclear speckles. Experiments were performed three times independently. Scale bar: 20 µm. (H) Western blot analysis of PHGDH protein levels in HNRNPU‐KO HeLa cells reconstituted with FLAG‐tagged wild‐type (WT) HNRNPU or the K181A, and subjected to the indicated manipulations (HA‐NAA50 overexpression and/or sodium L‐lactate treatment). (Original uncropped blots can be found in Supporting Information S2: “Original blots for Figure 8H.”) (I, J) Quantitative analysis of total PHGDH mRNA levels (I) and exon 1 (J) under the same conditions as in (H), as measured by qRT‐PCR. Data are presented as mean ± SD from three independent biological replicates (n = 3). Statistical significance was determined using one‐way ANOVA followed by Dunnett's multiple comparisons test. **p < 0.01, ****p < 0.0001. (K) RT‐PCR analysis of PHGDH exon 1–associated PHGDH transcript usage in HNRNPU‐KO HeLa cells reconstituted with HNRNPU WT or HNRNPU K181A and treated as indicated. Representative RT‐PCR results are shown in the lower panel, indicating exon 1–containing (+exon1) and exon 1–lacking (−exon1) transcripts. Quantification of PHGDH exon 1‐containing transcript ratio is shown in the upper panel. Data are presented as mean ± SD from three independent biological replicates (*n* = 3). Statistical significance was determined using one‐way ANOVA followed by Dunnett's multiple comparisons test. ***p < 0.001. (L) Actinomycin D chase assay showing the decay curves of PHGDH mRNA at 0, 4, 8, and 12 h under the indicated conditions, reflecting changes in mRNA stability. Data are presented as mean ± SD (*n* = 9). PHGDH mRNA decay curves were analyzed using two‐way repeated‐measures ANOVA, followed by Sidak's multiple‐comparisons test. Only selected pairwise comparisons at the 12 h time point are shown for clarity. ***p < 0.001, ****p < 0.0001.

Given the reduction in exon 1‐containing PHGDH transcripts under the HNRNPU K181A condition, we next asked whether HNRNPU K181A also affects PHGDH mRNA stability. Upon transcriptional inhibition using actinomycin D, PHGDH mRNA stability was found to be significantly reduced by HNRNPU K181A compared to control groups (Figure 8A). Previously reported HNRNPU consensus binding sequences [37] were analyzed to identify specific binding motifs on PHGDH mRNA (Figure 8B). Based on these findings, binding sites adjacent to PHGDH exon 1 were precisely mapped, and site‐specific mutants of the predicted HNRNPU‐binding motif were generated (Figure 8C). Notably, mutation of the HNRNPU‐binding motif markedly disrupted the interaction between HNRNPU protein and PHGDH mRNA (Figure 8D). Furthermore, mutation of this binding site abolished the HNRNPU K181A‐associated reduction in exon 1‐containing PHGDH transcripts, supporting the specificity of HNRNPU binding in regulating PHGDH RNA processing (Figure 8E). Nuclear speckles have been implicated in the post‐transcriptional regulation of gene expression, particularly in pre‐mRNA splicing [14]. To investigate the effect of HNRNPU lactylation on nuclear speckle organization, we performed immunofluorescence assays using an anti‐SON antibody, a specific marker for nuclear speckles, in cells expressing HNRNPU WT or the K181A mutant. The results showed that the wild‐type HNRNPU group exhibited relatively small and dispersed nuclear speckles, whereas expression of the K181A mutant led to larger and more aggregated SON‐positive speckles (Figure 8F). This phenotype may reflect coalescence and reorganization of nuclear speckles, accompanied by altered spatial compartmentalization of RNA‐processing factors, which may contribute to the alternative splicing abnormalities observed after HNRNPU K181A expression. To further assess whether HNRNPU lactylation influences nuclear speckle organization and the distribution of RNA‐processing factors, we performed L‐lactate treatment in cells expressing wild‐type HNRNPU. The results showed that, compared with the control group, L‐lactate treatment led to more dispersed and finer SON‐positive nuclear speckles (Figure 8G). These results suggest that enhanced HNRNPU lactylation may remodel nuclear speckle organization and alter the spatial compartmentalization of RNA‐processing factors. Together, these findings support a role for HNRNPU K181 lactylation in regulating nuclear speckle organization and the spatial compartmentalization of RNA‐processing factors.

To further examine the contribution of K181 modification and its interplay with acetylation and lactylation, we reconstituted HNRNPU‐KO HeLa cells with FLAG‐tagged wild‐type HNRNPU (WT) or the K181A mutant, and in the WT‐reconstituted background modulated the modification balance at this residue by overexpressing NAA50 or supplementing with sodium L‐lactate. In cells reconstituted with HNRNPU‐WT, NAA50 overexpression, which is expected to shift the modification balance toward acetylation at K181, significantly reduced PHGDH protein levels, whereas lactate treatment, which is expected to enhance lactylation at K181, markedly increased PHGDH expression. Co‐treatment with NAA50 and lactate partially restored PHGDH protein expression, consistent with an antagonistic interplay between acetylation and lactylation at K181. In parallel, cells reconstituted with the K181A mutant, included as a site‐mutant control, exhibited a PHGDH expression pattern distinct from that observed in the WT background, further supporting the involvement of K181 in this regulatory process (Figure 8H). At the RNA level, NAA50 overexpression similarly reduced total PHGDH mRNA abundance (Figure 8I), decreased the abundance of exon 1‐containing transcripts (Figure 8J), and increased exon 1‐related splicing alterations (Figure 8K), whereas lactate treatment produced the opposite effects. The combined treatment group exhibited intermediate phenotypes, further supporting a coordinated effect of competing modifications at K181 on PHGDH RNA processing and expression. To determine whether these opposing modifications also affect PHGDH mRNA stability, we performed actinomycin D chase assays and monitored PHGDH mRNA decay at 0, 4, 8, and 12 h. Two‐way repeated‐measures ANOVA revealed significantly different PHGDH mRNA decay kinetics among groups. In the HNRNPU‐WT background, NAA50 overexpression accelerated PHGDH mRNA decay, whereas lactate treatment delayed decay; the combined treatment group displayed an intermediate decay pattern. Consistent with its reduced transcript abundance, the K181A mutant also exhibited decreased PHGDH mRNA stability compared with the WT group (Figure 8L). Collectively, these results support a model in which the modification balance at HNRNPU K181 contributes to the regulation of PHGDH RNA processing, mRNA stability, and expression, with acetylation and lactylation exerting opposing effects.

### 2.7 PHGDH Is Essential for HNRNPU K181 Lactylation‐mediated Cervical Cancer Proliferation Both In Vitro and In Vivo

To evaluate the role of PHGDH‐mediated serine metabolism in HNRNPU K181lac‐regulated cell proliferation, PHGDH was overexpressed in HeLa KO‐HNRNPU cells stably expressing the HNRNPU K181A (Figure 9A). Rescue experiments indicated that PHGDH overexpression reversed the HNRNPU K181A‐induced reductions in intracellular serine levels, the NADPH/NADP^+^ ratio, GSH content, and the GSH/GSSG ratio (Figure 9B–E). Accordingly, the reductions in cell proliferation and clonogenic ability caused by HNRNPU K181A were effectively restored (Figure 9F,G). PHGDH overexpression also reversed the elevated ROS accumulation and reduced DNA synthesis capacity associated with the K181A mutation (Figure 9H,I). Furthermore, in vivo xenograft experiments confirmed that PHGDH mediates the tumor‐promoting effects of HNRNPU K181lac in cervical cancer proliferation (Figure 9J–L). Intriguingly, analysis of the xenograft tissues revealed a correlative increase in HNRNPU protein levels upon PHGDH overexpression (Figure 9K). This observation, coupled with emerging reports on the non‐metabolic functions of PHGDH in processes such as chromatin remodeling and translation initiation [38, 39], prompted us to hypothesize a potential reciprocal regulation. To test this, we examined whether PHGDH itself could regulate HNRNPU expression. Strikingly, overexpression of PHGDH in cervical cancer cells significantly upregulated both the mRNA and protein levels of endogenous HNRNPU (Figure 9M,N). These findings suggest that PHGDH may, in turn, influence HNRNPU expression. In the context of our study, this raises the possibility that HNRNPU lactylation promotes PHGDH expression, while elevated PHGDH may reciprocally enhance HNRNPU expression, thereby forming a potential positive feedback loop within this axis. However, whether this represents a direct regulatory circuit and its underlying molecular mechanism remain to be further investigated.

FIGURE 9PHGDH restoration rescues HNRNPU K181 lactylation‐mediated metabolic and proliferative effects in cervical cancer. (A) Western blot analysis of PHGDH overexpression in KO‐HNRNPU HeLa cells expressing HNRNPU K181A. Representative images from three independent biological replicates are shown. (Original uncropped blots can be found in Supporting Information S2: “Original blots for Figure 9A.”) (B–E) Metabolic parameters in KO‐HNRNPU HeLa cells expressing HNRNPU WT, HNRNPU K181A, or HNRNPU K181A with PHGDH overexpression: (B) Intracellular serine levels. (C) NADPH/NADP^+^ ratio calculated from enzymatic assays. (D) Total GSH content determined by colorimetric assay. (E) GSH/GSSG ratio. The X‐axis labels correspond to the following groups: Group 1, HNRNPU WT; Group 2, HNRNPU K181A; Group 3, HNRNPU K181A + Vector; Group 4, HNRNPU K181A + OEPHGDH. Data are presented as mean ± SD from three independent biological replicates (*n* = 3). Statistical significance was determined using one‐way ANOVA followed by Tukey's multiple comparisons test. ns, not significant; **p < 0.01, ***p < 0.001. (F) Cell proliferation measured by CCK‐8 assay at indicated time points. Data are presented as mean ± SD (*n* = 4 per group). Overall growth curves were analyzed using two‐way repeated‐measures ANOVA with group and time as factors. Statistical significance indicated in the graph was determined at the final time point (day 5) using one‐way ANOVA followed by Tukey's multiple comparisons test. ns, not significant; *p < 0.05. (G) Colony formation assay. Representative images from three independent biological replicates are shown (left). Quantification of colony numbers is shown in the right panel. The X‐axis labels correspond to the following groups: Group 1, HNRNPU WT; Group 2, HNRNPU K181A; Group 3, HNRNPU K181A + Vector; Group 4, HNRNPU K181A + OEPHGDH. Data are presented as mean ± SD (*n* = 3 per group). Statistical analysis was performed using one‐way ANOVA followed by multiple‐comparisons testing where appropriate. ns, not significant, ***p < 0.001. (H) Intracellular ROS levels detected by DCFH‐DA fluorescence microscopy. Representative images from three independent biological replicates are shown (left). Scale bar: 50 µm. Quantification of relative ROS levels is shown in the right panel. The X‐axis labels correspond to the following groups: Group 1, HNRNPU WT; Group 2, HNRNPU K181A; Group 3, HNRNPU K181A + Vector; Group 4, HNRNPU K181A + OEPHGDH. Data are presented as mean ± SD from three independent experiments. Statistical significance was determined using one‐way ANOVA followed by Tukey's multiple comparisons test. **p < 0.01, ***p < 0.001, ns: not significant. (I) DNA synthesis assessed by EdU incorporation assay. Representative images from three independent biological replicates are shown (left). Scale bar: 100 µm. Quantification of EdU‐positive cells is shown in the bottom panel. The X‐axis labels correspond to the following groups: Group 1, HNRNPU WT; Group 2, HNRNPU K181A; Group 3, HNRNPU K181A + Vector; Group 4, HNRNPU K181A + OEPHGDH. Data are presented as mean ± SD from three independent biological replicates (*n* = 3). Statistical significance was determined using one‐way ANOVA followed by Tukey's multiple comparisons test. ns, not significant, **p < 0.01, ***p < 0.001. (J) Xenograft tumor analysis. Tumor volume growth curves were measured at the indicated time points. Representative images of excised tumors from each group at day 24 are shown (n = 6 mice per group). Overall tumor growth curves were analyzed using two‐way repeated‐measures ANOVA with group and time as factors. Statistical significance indicated in the growth curves was determined at the final time point (day 24) using one‐way ANOVA followed by Tukey's multiple comparisons test. Tumor weights were analyzed using one‐way ANOVA followed by multiple‐comparisons testing where appropriate. The X‐axis labels correspond to the following groups: Group 1, HNRNPU WT; Group 2, HNRNPU K181A; Group 3, HNRNPU K181A + Vector; Group 4, HNRNPU K181A + OEPHGDH. ns, not significant, **p < 0.01, ***p < 0.001. (K, L) Representative images of HE staining and IHC staining of HNRNPU, Ki67, and PHGDH in xenograft tumor sections from the indicated groups. Scale bar: 50 µm. Quantification of HNRNPU‐, Ki67‐, and PHGDH‐positive cells (percentage of positive cells) is shown in panel (L). The X‐axis labels correspond to the following groups: Group 1, HNRNPU WT; Group 2, HNRNPU K181A; Group 3, HNRNPU K181A + Vector; Group 4, HNRNPU K181A + OEPHGDH. Data are presented as mean ± SD (*n* = 6 mice per group), with each data point representing one mouse. Statistical significance was determined using one‐way ANOVA followed by Tukey's multiple comparisons test. ns, not significant; **p < 0.01, ***p < 0.001. (M) qRT‐PCR analysis of HNRNPU mRNA levels in cervical cancer cells following PHGDH overexpression. Data are presented as mean ± SD from three independent biological replicates, each performed with three technical replicates. Statistical significance was determined by a two‐tailed unpaired *t*‐test. ****p < 0.0001. N) Western blot analysis of HNRNPU protein levels in cervical cancer cells following PHGDH overexpression. Representative images from three independent biological replicates are shown. (Original uncropped blots can be found in Supporting Information S2: “Original blots for Figure 9N.”).

**Figure:**
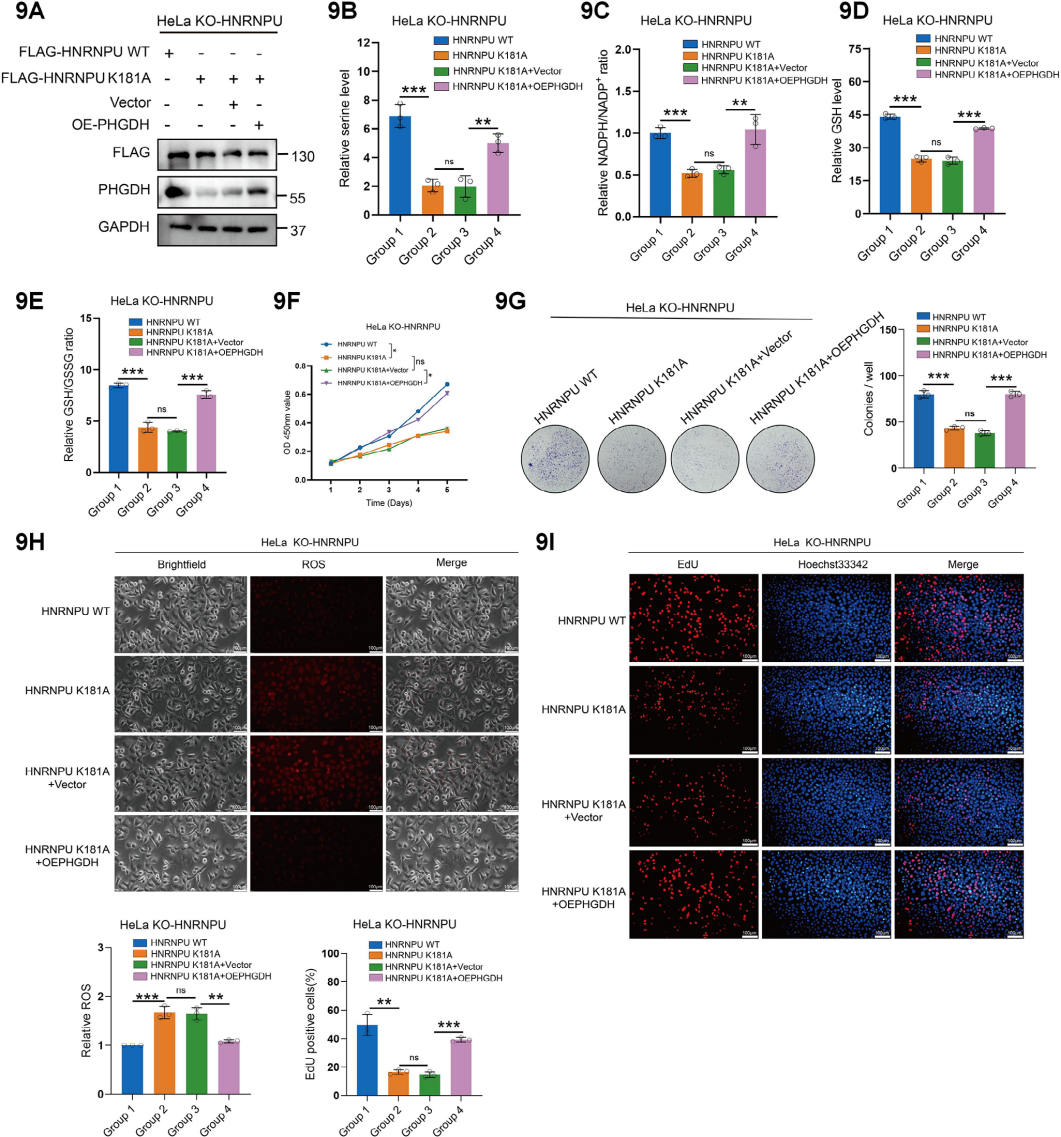

**Figure:**
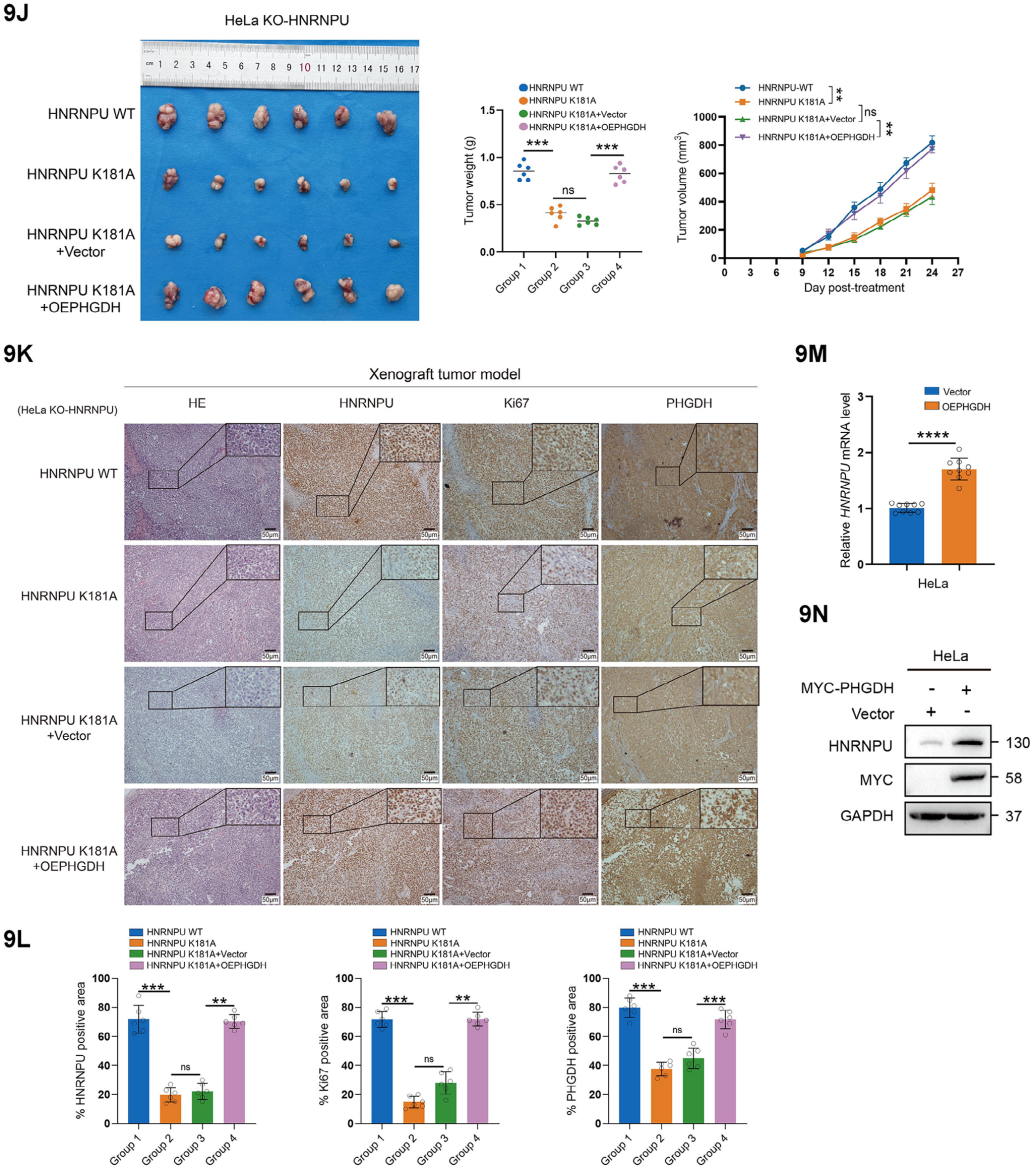

### 2.8 Pazopanib Inhibits Cervical Cancer Proliferation Through Suppression of HNRNPU K181 Lactylation

**FIGURE 10:**
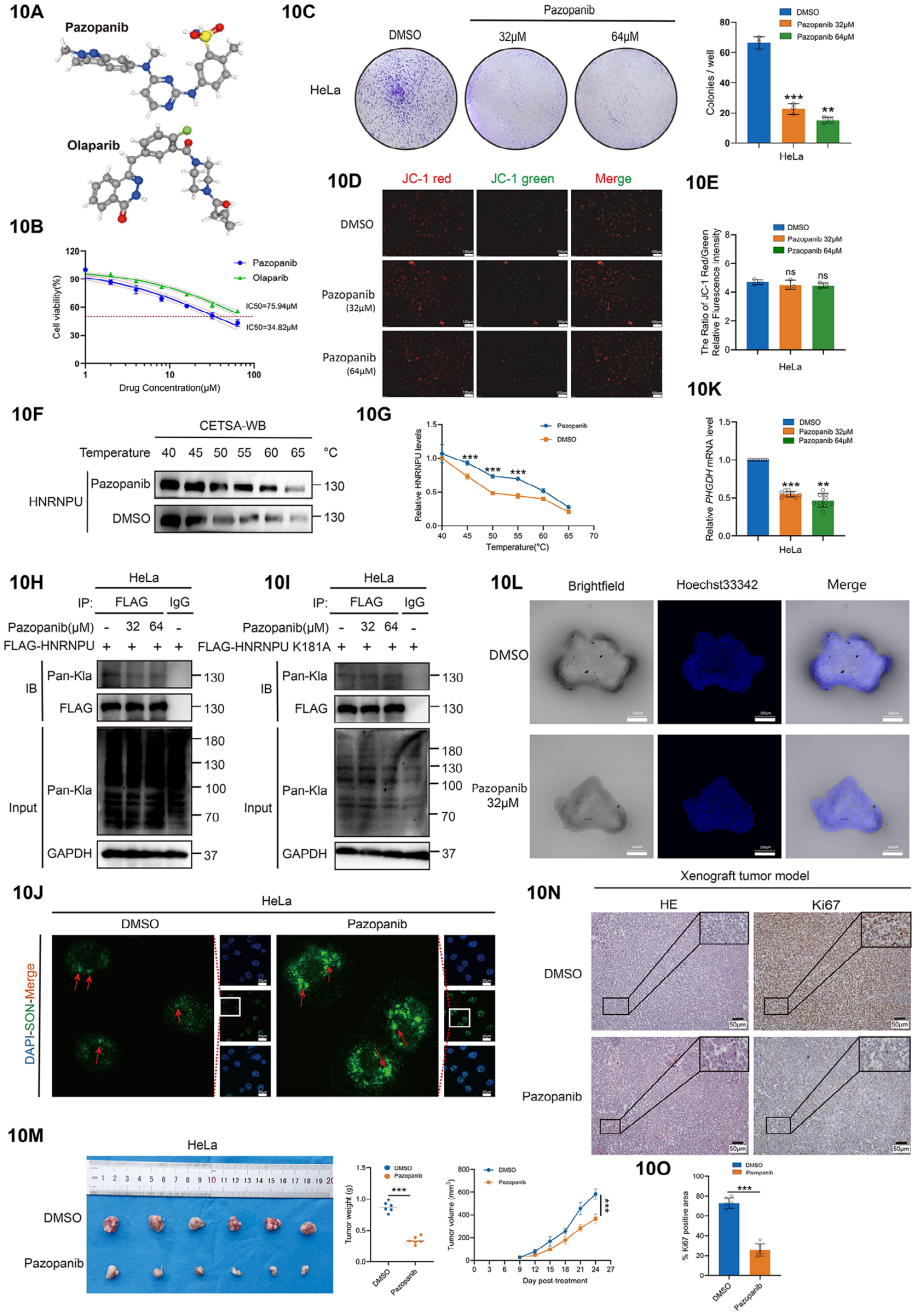
Pazopanib targeting HNRNPU K181 lactylation suppress cervical cancer proliferation. (A) Chemical structures of Pazopanib and Olaparib, two FDA‐approved compounds selected based on their documented anti‐tumor activity in cervical cancer. (B) Dose‐response curves of Pazopanib and Olaparib in HeLa cells (CCK‐8 assay, 24 h). Calculated IC_50_ values shown. Data are presented as mean ± SEM from three independent biological replicates, each performed with three technical replicates. (C) Colony formation assay of lactate‐treated HeLa cells with increasing Pazopanib concentrations (32 and 64 µM, 24 h). Representative images from three independent biological replicates are shown (left). Quantification of colony numbers is shown in the right panel. Data are presented as mean ± SD from three independent experiments. Statistical significance was determined by one‐way ANOVA with Tukey's multiple comparison test. **p < 0.01, ***p < 0.001. (D, E) JC‐1 assay showing mitochondrial membrane potential in Pazopanib‐treated cells (32 and 64 µM, 24 h). Red/green fluorescence ratio indicates viability. Representative images from three independent biological replicates are shown (left). Quantification of the red/green fluorescence ratio is shown in the right panel. Data are presented as mean ± SD from three independent experiments. Statistical significance was determined by one‐way ANOVA with Tukey's multiple comparison test. ns: not significant. (F) CETSA‐WB analysis of HNRNPU thermal stability in HeLa cells treated with 32 µM pazopanib or 0.1% DMSO for 1 h. Cell lysates were heated at the indicated temperatures, and soluble HNRNPU was detected by immunoblotting. Representative images from three independent biological replicates are shown. (Original uncropped blots can be found in Supporting Information S2: “Original blots for Figure 10F.”) (G) Quantification of relative HNRNPU levels from three independent CETSA experiments. Data are presented as mean ± SD (*n* = 3). Statistical analysis was performed using two‐way ANOVA with treatment and temperature as factors, followed by Tukey's multiple comparisons test. ***p < 0.001. (H) Western blot analysis of HNRNPU lactylation in Pazopanib‐treated HeLa cells. Representative images from three independent biological replicates are shown. (Original uncropped blots can be found in Supporting Information S2: “Original blots for Figure 10H.”) (I) Western blot confirming Pazopanib does not affect HNRNPU K181A mutant lactylation. Representative images from three independent biological replicates are shown. (Original uncropped blots can be found in Supporting Information S2: “Original blots for Figure 10I.”) (J) Representative immunofluorescence images of SON staining in HeLa cells treated with DMSO or Pazopanib (32 µM, 24 h). Compared with DMSO‐treated controls, Pazopanib treatment led to larger and more aggregated SON‐positive nuclear speckles. Experiments were repeated three times. Scale bar: 20 µm. (K) qRT‐PCR analysis of PHGDH mRNA in Pazopanib‐treated cells (32 and 64 µM, 24 h). Data are presented as mean ± SD from three independent biological replicates, each performed with three technical replicates. Statistical significance was determined by one‐way ANOVA with Tukey's multiple comparison test. **p < 0.01, ***p < 0.001. (L) Tumor spheroid formation assay with Pazopanib treatment (32 µM). Representative images and nuclear staining. Scale bar: 200 µm. M) In vivo antitumor efficacy of pazopanib in a xenograft model. Tumor volumes were measured at the indicated time points. Representative images of excised tumors are shown (left). Tumor weights at day 24 are shown in the middle panel. Data are presented as mean ± SD (*n* = 6 per group). Overall tumor growth curves were analyzed using two‐way repeated‐measures ANOVA with treatment and time as factors. Statistical significance indicated in the growth curves was determined at the final time point (day 24) using a two‐tailed unpaired t‐test. Tumor weights at day 24 were analyzed using a two‐tailed unpaired t‐test. ***p < 0.001. (N) Representative images of HE staining and IHC staining of Ki67 in xenograft tumor sections from DMSO‐treated and Pazopanib‐treated groups. Scale bar: 50 µm. (O) Quantification of Ki67‐positive cells (percentage of positive cells) in xenograft tumor sections is shown. Data are presented as mean ± SD (*n* = 6 mice per group), with each data point representing one mouse. Statistical significance was determined by a two‐tailed unpaired *t*‐test. ***p < 0.001.

Based on our finding that HNRNPU K181 lactylation promotes cervical cancer progression, we next explored whether clinically relevant pharmacological agents might modulate HNRNPU‐associated functions. Through literature review, we focused on two FDA‐approved compounds with documented anti‐tumor activity in cervical cancer: Pazopanib and Olaparib (Figure 10A). Pazopanib, a multi‐kinase inhibitor, has been evaluated in a randomized phase II clinical trial involving 230 patients with advanced or recurrent cervical cancer, demonstrating significant improvement in progression‐free and overall survival [40]. Olaparib, a PARP inhibitor, has shown preclinical efficacy against primary cervical cancer cell lines and significantly impairs xenograft tumor growth in vivo [41]. Given their established anti‐tumor activities, we sought to examine whether modulation of HNRNPU‐associated lactylation might be linked to their cellular effects. It should be noted that any such contribution would likely represent only a component of their broader mechanisms of action. We first assessed their effects on cell proliferation. The half‐maximal inhibitory concentrations (IC_50_) of Pazopanib and Olaparib in HeLa cells were preliminarily determined using CCK‐8 assays. Pazopanib demonstrated a significantly lower IC_50_ (34.82 µM) than Olaparib (75.94 µM), suggesting a stronger antiproliferative effect under our experimental conditions (Figure 10B). Of note, the steady‐state total plasma concentrations of pazopanib in patients receiving the standard 800 mg daily dose have been reported to range from approximately 30 to 50 µg/mL (65–115 µM), indicating that the concentrations used in our study (32–64 µM) are comparable to the reported range of total plasma concentrations. It should be noted that pazopanib is highly protein‐bound in plasma, and therefore the free drug concentration may be lower than total plasma levels. Pazopanib inhibited colony formation in a concentration‐dependent manner (Figure 10C). To exclude the possibility that the antitumor effects of Pazopanib are attributed to its cytotoxicity, post‐treatment analyses were performed. JC‐1 fluorescence analysis showed that Pazopanib treatment did not cause mitochondrial dysfunction (Figure 10D,E), supporting that its antitumor effects stem from antiproliferative rather than cytotoxic mechanisms. Based on the IC_50_ value, we selected 32 µM Pazopanib to determine whether it directly binds to HNRNPU by performing Cellular Thermal Shift Assay coupled with Western blot (CETSA‐WB). HeLa cells were treated with 32 µM Pazopanib or 0.1% DMSO for 1 h, and cell lysates were subjected to a temperature gradient ranging from 40°C to 65°C. As shown in Figure 10F, HNRNPU protein levels gradually decreased with increasing temperature in the DMSO control group, indicating temperature‐dependent thermal denaturation. Notably, Pazopanib treatment markedly shifted the melting curve of HNRNPU to the right, with significantly higher levels of soluble HNRNPU detected at 50°C and 55°C compared to the DMSO control. Quantification from three independent experiments confirmed that Pazopanib significantly enhanced HNRNPU thermal stability at these temperatures (Figure 10G). These results suggest that Pazopanib engages HNRNPU in a cellular context, consistent with an interaction between Pazopanib and HNRNPU. Pazopanib treatment (32 and 64 µM) partially inhibited HNRNPU lactylation in HeLa (Figure 10H), while had no effect on lactylation of the HNRNPU K181A mutant (Figure 10I). To determine whether this effect reflects a general regulation of lysine acylation, we examined HNRNPU acetylation under the same conditions using pan‐Kac antibodies. Notably, Pazopanib treatment did not result in detectable changes in the acetylation level of HNRNPU in either wild‐type or K181A mutant cells (Figure S6A,B). These findings suggest that Pazopanib does not broadly affect lysine acetylation on HNRNPU, supporting the specificity of its effect on lactylation. Given that HNRNPU K181 lactylation is associated with changes in nuclear speckle organization and splicing‐related nuclear architecture (Figure 8F,G), we next examined whether Pazopanib treatment affects nuclear speckle organization. Immunofluorescence staining using an anti‐SON antibody revealed that Pazopanib treatment (32 µM, 24 h) altered nuclear speckle morphology, with SON‐positive speckles becoming larger and more aggregated compared to DMSO‐treated controls (Figure 10J). This phenotype closely resembles that observed with the K181A mutant (Figure 8F), further supporting that Pazopanib inhibits HNRNPU lactylation at this site and consequently disrupts normal nuclear speckle organization. As anticipated, Pazopanib treatment significantly reduced PHGDH mRNA expression (Figure 10K). These findings support that Pazopanib inhibits cervical cancer cell proliferation, at least in part, through modulation of HNRNPU lactylation. To assess its efficacy in a physiologically relevant model, HeLa cell‐derived tumor spheriods were treated with Pazopanib, resulting in significant inhibition of spheroid formation (Figure 10L). Similarly, intraperitoneal administration of Pazopanib markedly inhibited xenograft tumor progression in nude mice (Figure 10M–O). These findings indicate that Pazopanib suppresses cervical cancer cell proliferation across multiple experimental models. Given that Pazopanib is a well‐established multi‐kinase inhibitor, its antiproliferative effects are likely multifactorial; nevertheless, our results raise the possibility that modulation of HNRNPU lactylation may represent an additional contributing mechanism.

## 3 Discussion

This study identifies HNRNPU as a critical oncogenic RNA‐binding protein in cervical cancer, with its tumor‐promoting function dependent on lysine lactylation at residue K181. L lactate, abundant in the tumor microenvironment, was shown to directly induce lactylation at K181, stabilizing HNRNPU and promoting cervical cancer cell proliferation. These findings support recent evidence that lactate functions not only as a metabolic byproduct but also as a signaling molecule mediating post translational modifications in key proteins [42, 43].

Mechanistically, lactylation at K181 enhances HNRNPU binding to PHGDH mRNA, supports maintenance of the exon 1‐containing PHGDH transcript, and is associated with increased PHGDH mRNA stability, ultimately promoting PHGDH upregulation. Additionally, a potential binding motif for HNRNPU was identified in exon 1 of PHGDH. After mutating this motif, the interaction between HNRNPU and PHGDH was weakened, and the alteration in exon 1‐associated PHGDH transcript usage was attenuated. PHGDH plays a rate limiting role in the serine biosynthesis pathway, essential for nucleotide synthesis and redox homeostasis during rapid tumor proliferation [44, 45]. This delineates a direct link between lactate driven signaling and metabolic reprogramming via post transcriptional RNA regulation. Interestingly, NAA50 mediated acetylation at K181 competitively antagonizes lactylation, suggesting a mutually exclusive PTM switch that modulates HNRNPU function. Such actylation crosstalk has been described in histone regulation and chromatin dynamics [46, 47], but its extension to non‐histone contexts highlights a broader regulatory mechanism.

To comprehensively evaluate the regulatory mechanisms governing HNRNPU stability, we systematically examined multiple post‐translational modifications (PTMs), including lactylation, ubiquitination, and acetylation. Our findings suggest that lactylation may contribute to the upregulation and stabilization of HNRNPU in the high‐lactate microenvironment of cervical cancer. While the ubiquitin–proteasome pathway participates in HNRNPU degradation, as indicated by MG132 treatment (Figure 2D), our data further show that lactate exposure reduces the ubiquitination level of HNRNPU (Figure 2E). These observations suggest that lactylation may function upstream of ubiquitin‐mediated degradation and contribute to protein stability. However, we do not exclude the involvement of additional post‐translational modifications (PTMs), such as phosphorylation and methylation, in this process. In addition, our data indicate that NAA50‐mediated acetylation at K181 exhibits an inverse temporal relationship with lactylation at the same residue, and that these two modifications exert opposing effects on HNRNPU stability. Together with the identification of K181 as a shared modification site, these findings are consistent with a model in which distinct PTMs at the same lysine residue may functionally counteract each other. Similar crosstalk between lactylation and acetylation has been reported in recent studies [48, 49], suggesting that such interactions may represent a broader regulatory paradigm. Consistent with this model, in the HNRNPU‐WT‐reconstituted background, modulation of NAA50 expression and lactate exposure produced opposing effects on PHGDH protein expression, PHGDH mRNA abundance, and exon 1‐containing PHGDH transcript output, while combined treatment yielded intermediate phenotypes. In addition, actinomycin D chase assays showed that NAA50 overexpression accelerated PHGDH mRNA decay, whereas lactate delayed it, further supporting an antagonistic functional relationship between K181 acetylation and lactylation in the regulation of PHGDH. However, we note that our current data do not provide direct evidence of mutual exclusivity at the single‐molecule level or quantitative stoichiometric competition. Further studies using site‐specific modification mimetics, advanced proteomics, or high‐resolution imaging will be required to rigorously define this mechanism.

Our study reveals that lysine 181 (K181) of HNRNPU is subject to both acetylation and lactylation. This finding suggests that this residue may function as a regulatory node, enabling HNRNPU to respond to distinct intracellular signals. A central question arising from this discovery is whether these modifications occur stochastically or are differentially employed under specific physiological conditions to precisely modulate HNRNPU function. We propose that acetylation and lactylation at K181 may constitute a dynamic “functional switch”. Given that acetylation is a well‐characterized mechanism driving cell cycle progression, such as the G1/S transition [50], K181 acetylation might be predominant during active proliferation phases, thereby promoting HNRNPU's involvement in the mRNA metabolism of proliferation‐related genes. Conversely, under stress conditions like DNA damage, K181 lactylation is likely elevated. This notion is supported by the metabolic reprogramming and lactate accumulation frequently associated with the DNA damage response [51], coupled with the established role of lactylation as a key modification translating metabolic signals into epigenetic and gene regulatory commands [52]. Furthermore, HNRNPU itself has been demonstrated to maintain genome stability by promoting classical non‐homologous end joining (C‐NHEJ) [53]. Therefore, it is plausible that lactylated HNRNPU is specifically recruited to RNA metabolism pathways dedicated to DNA damage repair. These considerations outline a precise direction for future investigation: to validate the dynamics of K181 modifications and decipher their functional specificity. Quantitative analysis of modification changes at this site under cell cycle‐synchronized or specific metabolic conditions, combined with genetic approaches (e.g., constructing point‐mutant cell lines such as K181Q or K181R) to functionally decouple the two modifications, holds the promise of delineating the distinct pathways regulated by acetylation and lactylation, respectively. Ultimately, such work will elucidate how HNRNPU utilizes this sophisticated post‐translational modification switch to integrate upstream and downstream signals, thereby coordinating its diverse cellular functions.

To validate PHGDH as a key downstream effector, a lactylation deficient HNRNPU K181A mutant was constructed, which significantly impaired cell proliferation. Subsequent ectopic overexpression of PHGDH successfully rescued this proliferative defect, directly demonstrating PHGDH's indispensability in mediating the oncogenic effects of HNRNPU K181 lactylation.

Moreover, small‐molecule compounds were found to suppress cervical cancer cell growth both in vitro and in vivo, accompanied by modulation of HNRNPU K181 lactylation, suggesting that this post‐translational modification may be therapeutically relevant. While most therapeutic efforts to date have focused on histone lactylation, our findings raise the possibility that non‐histone lactylated proteins may also represent promising targets [54].

An intriguing observation from our rescue experiments was the correlative increase in HNRNPU levels upon PHGDH overexpression in vivo. Subsequent validation confirmed that PHGDH can upregulate endogenous HNRNPU expression, suggesting a potential positive feedback mechanism. While the precise molecular pathway requires further elucidation, this reciprocal relationship hints at a self‐reinforcing circuit that could potentiate the oncogenic output of the HNRNPU‐PHGDH axis. This possibility is consistent with reports of PHGDH's involvement in non‐metabolic processes such as chromatin remodeling, meriting future investigation into how its dual functions are integrated.

Despite these findings, the precise mechanism underlying the exon 1‐associated PHGDH transcript change remains unresolved. Our current data support that HNRNPU binds PHGDH RNA and is associated with maintenance of exon 1‐containing PHGDH transcripts, PHGDH mRNA stability, and PHGDH expression. Under the HNRNPU K181A condition, we observed a reduced proportion of exon 1‐containing PHGDH transcripts together with altered 5′‐end RNA processing patterns. However, because first exon‐associated changes may also arise from alternative promoter usage or alternative transcription start site selection [55], the present data are insufficient to distinguish among these possibilities or other exon 1‐associated RNA‐processing events [56]. Therefore, rather than defining this event as a fully established classical exon 1 skipping mechanism, we interpret our data more cautiously as evidence that HNRNPU helps maintain exon 1‐containing PHGDH transcript output and PHGDH mRNA stability. Future studies using approaches that directly resolve transcript initiation and 5′‐end architecture, such as 5′ RACE and CAGE‐seq, will be required to distinguish among alternative promoter usage, alt ernative transcription start site selection, and other exon 1‐associated RNA‐processing patterns in this region [57]. Second, the dynamic interplay between lactylation and acetylation at K181—particularly in response to fluctuating metabolic conditions within the tumor microenvironment—remains incompletely understood and requires further exploration. Third, although CETSA experiments confirmed direct binding of Pazopanib to HNRNPU, the precise binding site remains to be determined. The CETSA data demonstrate that Pazopanib stabilizes HNRNPU, and the K181A mutation abolishes its effect on lactylation, collectively suggesting that pazopanib may bind in the vicinity of K181. However, to precisely localize the binding site and resolve the atomic‐level interaction mode, further structural biology studies such as co‐crystallization or X‐ray crystallography are required. Furthermore, as a multi‐kinase inhibitor, Pazopanib's anti‐tumor effects in cervical cancer may involve synergistic effects of multiple targets. While we have demonstrated that inhibition of HNRNPU K181 lactylation is one of its newly discovered mechanisms, the relative contribution of this mechanism to overall efficacy remains to be quantified. Lastly, as a newly identified regulator of protein lactylation, NAA50 warrants further investigation to elucidate its biological functions—particularly whether it exclusively inhibits HNRNPU lactylation or also functions as a lactyltransferase for other lactylated substrates.

In conclusion, this work uncovers a novel lactate driven regulatory axis, whereby HNRNPU K181 lactylation enhances PHGDH expression through post‐transcriptional mechanisms, thereby promoting serine metabolic reprogramming and cervical cancer progression. Targeting this lactylation dependent axis may provide a novel therapeutic strategy against metabolically active malignancies.

## 4 Experimental Section

### 4.1 Lentivirus Production and Generation of Stable Cell Lines

To establish stable cell lines overexpressing HNRNPU, PHGDH, or the HNRNPU K181A mutant, lentiviral transduction was performed. Target cells were infected with recombinant lentivirus for 24 h, followed by antibiotic selection using either 2 µg/mL puromycin (HY‐B1743, MCE) or 200 µg/mL hygromycin B (HY‐B0490, MCE) for 7–10 days. Successful overexpression was verified by Western blot analysis using specific antibodies against the target proteins.

### 4.2 Cell Culture, Transfection, and Drug Treatment

HEK293T cells, normal cervical epithelial cells (HcerEpic), and cervical cancer cell lines (HeLa, C33A, SiHa) were maintained in Dulbecco's Modified Eagle Medium (DMEM; Dalian Meilun Biotechnology) supplemented with 10% fetal bovine serum (Fetal Bovine Serum, FBS(BaiDi Biotechnology Co., Ltd.(BDBIO)) at 37°C in a humidified 5% CO_2_ atmosphere. Pipette tips used for cell culture were purchased from EIKON HEALTH. For transfection experiments, cells at 50–60% confluence were transfected using Lipofectamine 2000 (Xancon Biotechnology Co., Ltd. (Hebei, China), CK012) according to the manufacturer's protocol. Briefly, plasmid DNA was diluted in serum‐free DMEM and mixed with Lipofectamine 2000 (ratio optimized for each cell line). The DNA‐lipid complexes were added to cells and incubated for 8 h before replacing with complete growth medium. The following constructs were obtained from GeneChem: FLAG‐tagged HNRNPU (wild‐type, K181A, and K247A mutants), HA‐tagged NAA50 (wild‐type and E41A mutant), shRNA targeting HNRNPU, and overexpression vectors for NAA50 and PHGDH. For pharmacological interventions: Lactate treatment: 25 mM sodium L‐lactate (NaLa; Merck, 71718) for 24 h. Glycolysis inhibitors: 2‐deoxyglucose (2‐DG; HY‐13966, MCE), dichloroacetate (HY‐Y0445A, MCE), and oxamate (HY‐W013032A, MCE) for 24 h. Proteasome inhibition: 10 µM MG‐132 (C3348, APExBIO). Targeted therapies: Pazopanib (HY‐10208, MCE) and olaparib (HY‐10162, MCE) at indicated concentrations for 24 h. All cell culture dishes and 20‐mm glass‐bottom dishes were obtained from NEST Biotechnology.

### 4.3 Animal Experiments

The Animal Research Ethics Committee of Jilin University approved all animal experiments. Female BALB/c nude mice (4‐6 weeks old) were purchased from Beijing HFK Bio‐Technology Company (Beijing, China) and and housed under specific pathogen‐free conditions with a 12‐hour light/dark cycle, controlled temperature (22 ± 1°C), and humidity (50 ± 5%). For tumor xenograft studies, HeLa or SiHa cells stably expressing the proteins of interest were established via lentiviral transduction. Cells (5 × 10^6^ in 100 µL PBS) were subcutaneously injected into the right flank of each mouse (*n* = 5–8 per group). Starting on day 10 or 12 after injection, tumor volumes were measured every 2 or 3 days using a calliper and calculated using the following equation: volume = width × depth × length × 0.5.

### 4.4 CCK8 and Colony Formation

Breast cancer cells were cultured in a six‐well plate and subsequently transfected with plasmids or drugs treated for a 24‐h. Following this, the cells underwent trypsinization, and cell density was assessed to determine the requisite number of cells for further experimentation. CCK8 analysis involved seeding 2000 cells per well in a 96‐well plate configuration. The characterization of the cells was conducted following the manufacturer's protocols, utilizing a CCK8 assay kit (BioTek Instruments). The optical density was recorded at 490 nm using a microplate reader at time points of 24, 48, 72, and 96 h. In the colony formation assay, 1000 cells were planted in each well of six‐well plates until cell clusters formed. Post‐rinsing the colonies twice with cold PBS, the cells were stained with 0.1% crystal violet and preserved in 75% ethanol for colony visualization. The colonies were subsequently counted and photographed.

### 4.5 EdU Incorporation Assay

EdU incorporation assays were performed using a Cell‐Light EdU Apollo 488 In Vitro Imaging Kit (Beyotime Company, Shanghai, China) according to the manufacturer's instructions. Images were captured using an Olympus DP70 microscope (Olympus), and the number of EdU‐positive cells was counted.

### 4.6 Western Blotting

Cells were lysed in RIPA lysis buffer supplemented with protease and phosphatase inhibitor cocktails. The protein concentration was determined using a BCA protein assay kit (KeygenBioTECH, KGB2101‐5000) according to the manufacturer's instructions. Protein samples were mixed with protein loading buffer (Biolinkedin, Shanghai, China, Cat. No. L‐7102) and boiled before SDS‐PAGE. Equal amounts of protein lysates were separated by SDS‐PAGE and transferred onto PVDF membranes. After blocking with 5% non‐fat milk or 5% bovine serum albumin at room temperature for 1 h, the membranes were incubated with the indicated primary antibodies overnight at 4°C. After washing with TBST, the membranes were incubated with HRP‐conjugated secondary antibodies at room temperature for 1 h. Protein bands were visualized using an enhanced chemiluminescence detection system.

The primary antibodies used for western blotting were as follows: anti‐HNRNPU antibody (Proteintech, Cat. No. 14599‐1‐AP), anti‐PHGDH antibody (Proteintech, Cat. No. 14719‐1‐AP), anti‐NAA50 antibody (ABclonal, Cat. No. A7387), anti‐AARS1 antibody (zenbio, Cat. No. 861254), anti‐pan‐lactyllysine antibody (Pan‐Kla; PTM BIO, Cat. No. PTM‐1425RM), anti‐pan‐acetyllysine antibody (Pan‐Kac; Proteintech, Cat. No. 66289‐1‐Ig), anti‐ubiquitin antibody (HUABIO, Cat. No. ET1609‐21), anti‐FLAG antibody (Proteintech, Cat. No. 66008‐4‐Ig), anti‐HA antibody (Proteintech, Cat. No. 51064‐2‐AP), anti‐Vinculin antibody (Santa cruz, sc‐73264) and anti‐GAPDH antibody (Proteintech, Cat. No. 60004‐1‐Ig). HRP‐conjugated anti‐rabbit IgG and anti‐mouse IgG secondary antibodies were purchased from Proteintech (Cat. Nos. RGAR001 and RGAM001).

### 4.7 Immunofluorescence

Cells, previously cultured on coverslips, were seeded 1 d before immunofluorescence analysis, reaching a final confluence of 70–80%. Glass‐bottom culture dishes used for immunofluorescence staining were purchased from Guangzhou Jet Bio‐Filtration Co., Ltd. The cells were fixed with 4% paraformaldehyde for 10 min, permeabilized using 0.1% Triton X‐100 for 5 min, blocked with 5% bovine serum albumin, and subsequently incubated with the specified antibodies. This was followed by staining with Texas Red‐conjugated anti‐rabbit IgG and fluorescein isothiocyanate‐conjugated anti‐mouse IgG. The cells were mounted with a DAPI‐containing medium (Helixgen), and images were captured using a microscope (Olympus). The antibodies used for immunofluorescence staining were as follows: anti‐SON antibody (Proteintech, Cat. No. 83787‐5‐RR), anti‐NAA50 antibody (ABclonal, Cat. No. A7387), and anti‐HNRNPU antibody (Santa Cruz Biotechnology, Cat. No. sc‐365852).

### 4.8 NADPH Measurements

Intracellular NADPH was measured using cell lysates with an NADPH assay kit (Beyotime, S0179) according to the manufacturer's instructions.

### 4.9 Intracellular ROS Measurement

Intracellular ROS levels were quantified using the fluorescent probe carboxy‐H2DCFDA (Invitrogen). Briefly, cells were seeded in 6‐well plates at a density of 2 × 10^5^ cells/well and allowed to adhere for 24 h. After PBS washing, cells were loaded with 5 µM carboxy‐H2DCFDA in serum‐free medium and incubated for 30 min at 37°C in the dark. Following three washes with PBS, the oxidation‐sensitive fluorescent signal was immediately visualized and captured using an inverted fluorescence microscope (Olympus).

### 4.10 Immunohistochemistry

Immunohistochemical staining was performed on formalin‐fixed, paraffin‐embedded cervical cancer tissue sections according to standardized protocols at the Histomorphology Platform of Jilin University. Tissue specimens were fixed in 4% paraformaldehyde for 24 h at room temperature, processed through graded ethanol series, and embedded in paraffin. Four‐micrometer sections were cut and mounted on charged slides. Following deparaffinization in xylene and rehydration through graded alcohols, antigen retrieval was performed using citrate buffer (pH 6.0) under high‐pressure heating (121°C for 3 min). Endogenous peroxidase activity was quenched with 3% hydrogen peroxide for 10 min at room temperature. Non‐specific binding was blocked with 5% normal goat serum for 30 min at 37°C. Primary antibody incubation was carried out overnight at 4°C in a humidified chamber. After three 5‐minute washes with PBS, sections were incubated with horseradish peroxidase (HRP)‐conjugated secondary antibody for 30 min at 37°C. Chromogenic development was performed using 3,3'‐diaminobenzidine (DAB) substrate for 3–5 min, followed by hematoxylin counterstaining. Slides were dehydrated through graded alcohols, cleared in xylene, and mounted with resinous medium. The staining index was based on the staining intensity, which was graded as “−,” no staining; “+,” weak staining; “++,” moderate staining; and “+++,” strong staining. Samples that scored as “−” or “+” were considered as low expression and those scored as “++” or “+++” as high expression. All stained slides were ob‐ served and scored by two pathologists. If the staining interpretation differed between the two investigators, the data for the slide were discarded. For determining the H score, stained tissues were scored by calculating the product of the intensity level and the percentage of cells staining at that level (0, negative; 1, weak; 2, moderate; 3, strong). An H score was then calculated by summing the individual intensity level scores.

### 4.11 LC‐MS for Lactylation Detection

HEK293T cells were transfected with the FLAG‐HNRNPU plasmid for 24 h and then exposed to L‐lactate for 24 h. Cell lysates were treated with 1% sodium dodecyl sulfate (SDS) before immunoprecipitation using an antibody targeting the His epitope tag. SDS‐PAGE was performed, followed by Coomassie blue staining and the excision of bands for detection. Mass spectrometry (LTQ Orbitrap Elite, Thermo Fisher Scientific) was used to identify the lactylation of the HNRNPU protein.

### 4.12 mRNA Stability Assay

The stability of PHGDH mRNA was evaluated using the transcriptional inhibition assay. Cells were treated with actinomycin D (ActD; HY‐17559, MCE) at a final concentration of 5 µg/mL to inhibit de novo transcription. At specific time points (0, 4, 8, and 12 h) following ActD treatment, total RNA was extracted from the cells using a appropriate method. The abundance of PHGDH mRNA at each time point was quantified by reverse transcription‐quantitative real‐time PCR (qPCR) using gene‐specific primers. The mRNA decay kinetics were analyzed by plotting the relative PHGDH mRNA levels (normalized to a stable reference gene) against the time after ActD treatment.

### 4.13 RNA Immunoprecipitation (RIP)

Immunoprecipitation targeting HNRNPU and PHGDH mRNAs was performed utilizing the RNA Immunoprecipitation (RIP) kit (Catalog Bes5101, BersinBio, China) in accordance with the manufacturer's instructions. Total RNA was extracted from the cells using trizol. The extracted RNA was subsequently reverse‐transcribed into complementary DNA (cDNA) using the Hifair II first Strand cDNA Synthesis Kit (11121ES60, Yeasen, China), following the manufacturer's protocol. Quantitative reverse transcription‐polymerase chain reaction (qPCR) was conducted using the Hieff qPCR SYBR Green Master Mix (11202ES03, Yeasen, China), and the analysis was performed on a 7300 Fast Real‐Time PCR system (ThermoFisher Scientific, USA). The relative enrichment of RNA was normalized to the input.

### 4.14 Quantitative Real‐Time PCR

Total RNA was extracted using the TRIzol method. Total RNA (500 ng) was selected and reverse‐transcribed using Hifair II first Strand cDNA Synthesis SuperMix (YEASEN #11120ES60). The cDNA was used for qualitative real‐time PCR using the NovoStart SYBR qPCR SuperMix Plus (Cat. No.:E166, Novoprotein, Shanghai, China). The melting curve was examined to verify the amplification of a single product. For quantitative analysis, all samples were normalized to ubiquitin C gene expression using the ΔΔCT value method. The primer sequences are listed in Table S3.

### 4.15 Real‐Time PCR

RNA was isolated from the cells employing the Trizol reagent, adhering meticulously to the protocol outlined by Invitrogen. Residual genomic DNA present in the total RNA extract (1 microgram) was thoroughly eliminated and the purified RNA was subsequently reverse transcribed into cDNA utilizing the Hifair II first Strand cDNA Synthesis Kit (11121ES60, Yeasen, China), with 1 microliter of the synthesized cDNA being utilized for subsequent polymerase chain reaction (PCR) amplification. The primers used are listed in Table S3.

### 4.16 Transcript Measurement

A total RNA extract of cervical cells was prepared using TRIzol reagent (15,596,026, Invitrogen, USA). The reverse transcription of RNA was performed using the Hifair II first Strand cDNA Synthesis Kit (11121ES60, Yeasen, China) as the manufacturer's instructions. Percentspliced in index Exon 1–containing transcript ratio was calculated using the formula: Exon 1–containing transcript ratio  =  PHGDH(+exon1) / (PHGDH(+exon1) + PHGDH(‐exon1)). In this study, the ratio value was used to estimate the relative abundance of the exon 1‐containing PHGDH transcript within the detected transcript pool, rather than to claim definitive classical first‐exon skipping. The usage of primers for RT‐PCR was as follows:

GAPDH forward, 5‐TGCACCACCAACTGCTTAG‐3, reverse, 5‐GATGCAGGGATGATGTTC‐3; PHGDH (+ exon1), forward, 5‐CCTTGGATTGGTCTGGCAGA‐3, reverse,5‐CATGTCCACGAACTGAACAG‐3;

PHGDH (‐exon1), forward, 5‐ACTATAGGGTATGACCCCAT‐3, reverse,5‐CATGTCCACGAACTGAACAG‐3;

PHGDH‐primer1, forward, 5‐GTGAATGCCCAGGCCCTTAC‐3, reverse,5‐CCTGTGTTATCACCTGGATG‐3;

PHGDH‐primer2, forward, 5‐GCAGATTCCCCAGGCGACGGCTTCGATGAA‐3, reverse,5‐CTTCTTCCGCTCCCATTTGCCGTCCTTCAT‐3;

PHGDH‐primer3, forward, 5‐CTACCAGACTTCACTGGTGT‐3, reverse,5‐GTACTACAGGGTCAGAGTGC‐3.

### 4.17 Reduced Glutathione (GSH) Measurement

Reduced glutathione (GSH) levels were measured using a Reduced Glutathione (GSH) Content Assay Kit (Boxbio, China; Cat. No. AKPR008M) according to the manufacturer's instructions. Briefly, cells were collected and lysed, and the supernatants were used for GSH detection following the kit protocol. The absorbance was measured using a microplate reader, and intracellular GSH levels were calculated according to the standard curve and normalized to protein concentration.

### 4.18 JC‐1 Assay

JC‐1 assay was performed according to the manufacturer's instructions (C2006, Beyotime, China).

### 4.19 LC‐MS Analysis of Cell Metabolites

Approximately 5 × 10^6^ cells were washed twice with cold PBS, and polar metabolites were immediately extracted with ice‐cold 80% methanol. Samples were subjected to freeze–thaw cycles or sonication to extract the metabolites. The supernatants were collected and dried. The powder containing metabolites was dissolved in 80% methanol to run LC‐MS. For the kinetic LC‐MS analyses, a Shimadzu Nexera × 2 UHPLC combined with a Sciex 5600 Triple Time of Flight‐Mass Spectrometry (TOFMS) was used, which was controlled by Sciex Analyst 1.7.1 instrument acquiring software. A Supelco Ascentis Express HILIC Acquity UPLC BEH Amide (150 cm × 2.1 mm, 1.7 µm) column was used with mobile phase (A) consisting of 5 mM ammonium formate and 0.05% formic acid; mobile phase (B) consisting of 90% acetonitrile (ACN) and 10% water. Gradient program: mobile phase (A) was held at 15% for 0 min and then increased to 30% in 7 min; then to 60% in 6 min and held for 1 min before returning initial condition. The column was held at 40°C and 5 µL of sample was injected into the LC‐MS with a flow rate of 0.2 mL/min. Automatic calibrations of TOFMS were achieved with average mass accuracy of <2 ppm. Data Processing Software included Sciex PeakView 2.2, MasterView 1.1, and MultiQuant 3.0.2.

### 4.20 Molecular Docking and Virtual Screening

The protein structure was retrieved from UniProt and the full‐length AlphaFold predictive structure of HNRNPU (UniProt ID: Q00839) was selected as the ligand‐protein, while the full‐length AlphaFold predicted structure of NAA50 (UniProt ID: Q9GZZ1) was selected as the receptor protein. Alphafold3 was used for dock analysis, five interaction models were predicted. Model with the best ranking score was selected to analyze the predicted interaction sites, and PyMOL Software 2.6 was used to map the specific interaction amino acid sites. For virtual screening, centering on site 181 lysine at HNRNPU protein, the binding region was enlarged to the maximum to determine its active pocket. Docking analysis with HNRNPU active pockets was performed using a commercialized library of drug compounds (FDA‐approved Drug Library).

### 4.21 Statistical Analysis

All statistical analyses were performed using GraphPad Prism 10 (GraphPad Software, USA). Data are presented as mean ± standard error of standard deviation (SD), as specified in the corresponding figure legends. Comparisons between two groups were performed using an unpaired two‐tailed Student's t‐test. For analyses involving three or more groups, one‐way ANOVA followed by an appropriate multiple comparison test was used. When equal variances were not assumed, Welch's one‐way ANOVA followed by Dunnett T3 multiple comparisons test was applied. For data that did not meet normality assumptions, nonparametric tests were used. Statistical significance was defined as *p* < 0.05 and indicated as follows: *p* < 0.05 (*), *p* < 0.01 (**), *p* < 0.001 (***), *p* < 0.0001 (****). Sample sizes (n) indicated in the Figure legends represent biological replicates. No data points were excluded unless otherwise stated. All experiments were independently repeated at least three times, unless otherwise specified.

## Author Contributions

Z.C. and M.Q.F. designed the study. Z.C. conducted the experiments and wrote the manuscript. J.H., L.H.X., and W.X.C analyzed the data and directed the details of the experiment. Z.H.L and W.Y.S. provided funding support. All authors have reviewed and approved the paper.

## Funding

This research was supported by the National Natural Science Foundation of China (No. 82470786 to Yishu Wang; No. 82270785 to Honglan Zhou), the Talent Reserve Program of The First Hospital of Jilin University (JDYY‐TRP‐2026002), and the Graduate Innovation Fund of Jilin University (2026CX255).

## Disclosure

The authors declare that they have no known competing financial interests or personal relationships that could have appeared to influence the work reported in this paper.

## Ethics Statement

All specimens were collected as part of standard clinical care rather than being prospectively acquired for research purposes. The study protocol was approved by the Institutional Ethics Committee of the First Hospital of Jilin University (Approval No. 2021–315). Written informed consent was obtained from all participants. All animal experiments were approved by the Ethics Committee of the College of Basic Medical Sciences, Jilin University (Approval No. 2025–647) and performed in strict accordance with ARRIVE guidelines. All the above experiments were conducted in accordance with the Helsinki Declaration.

## Consent

All co‐authors have read and approved the final version of the manuscript and its submission to this journal.

## Conflicts of Interest

The authors declare no conflicts of interest.

## Supporting information




**Supporting File 1**: advs75866‐sup‐0001‐SuppMat.docx.

**Supporting File 2**: advs75866‐sup‐0002‐blotts.zip.

**Supporting File 3**: advs75866‐sup‐0003‐TableS1.xlsx.

**Supporting File 4**: advs75866‐sup‐0004‐TableS2.xlsx.

## Data Availability

The data that support the findings of this study are available from the corresponding author upon reasonable request. The RNA‐seq data generated in this study have been deposited in the Gene Expression Omnibus (GEO) under accession number GSE328486. The mass spectrometry proteomics data have been deposited to the ProteomeXchange Consortium via the PRIDE repository under accession number PXD077071.
