# Supplementary material for: HNRNPU K181 Lactylation Drives Cervical Cancer Growth by Upregulating PHGDH and Reprogramming Serine Metabolism
Source: Adv Sci (Weinh). 2026 May 27:e20222. Online ahead of print. doi: 10.1002/advs.202520222 (PMC13336110; doi:10.1002/advs.202520222)
Supplement: Supplementary file 1 — Supporting File 1: advs75866‐sup‐0001‐SuppMat.docx. [file ADVS-9999-e20222-s004.docx]

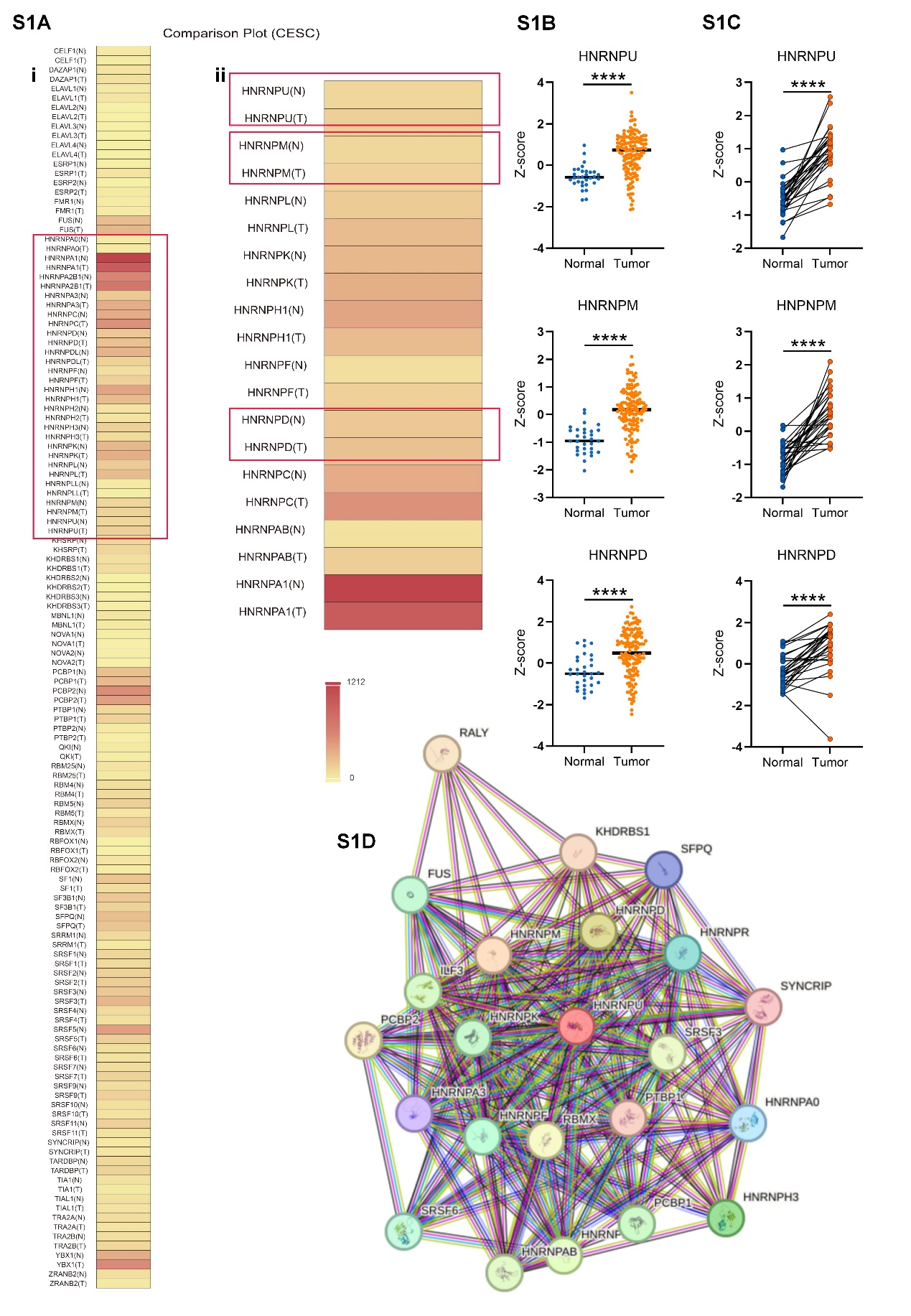


**Fig.S1 Systematic screening of splicing regulators and network analysis in cervical cancer.** A) Expression analysis of splicing factor families and HNRNP members in cervical cancer. (i) Comparison of overall expression levels among major splicing factor families in CESC from the GEPIA database. (ii) mRNA expression of HNRNPU, HNRNPM, and HNRNPD in tumor versus normal tissues from the TCGA-CESC cohort. B) Scatter dot plots showing the Z‑score of protein abundance for HNRNPU, HNRNPM, and HNRNPD in cervical cancer tissues (Tumor, n=136) and normal cervical tissues (Normal, n=33) from the PRIDE dataset (PXD055203). Each dot represents an individual sample. Statistical significance was assessed by two-tailed unpaired t-test. *****P* < 0.0001. C) Paired scatter dot plots showing the Z‑score of protein abundance for HNRNPU, HNRNPM, and HNRNPD in matched pairs of cervical cancer (Tumor) and adjacent normal tissues (Normal) from the PRIDE dataset (PXD055203). Each pair of connected dots represents one of the 30 paired samples. Statistical significance was assessed by paired Student’s t‑test. *****P* < 0.0001. D) Protein–protein interaction network of HNRNP family candidates. The network was constructed using STRING (confidence score > 0.7) and includes HNRNPU, HNRNPM, HNRNPD (highlighted in red) and their directly interacting partners. Node size corresponds to degree, and color intensity reflects betweenness centrality.


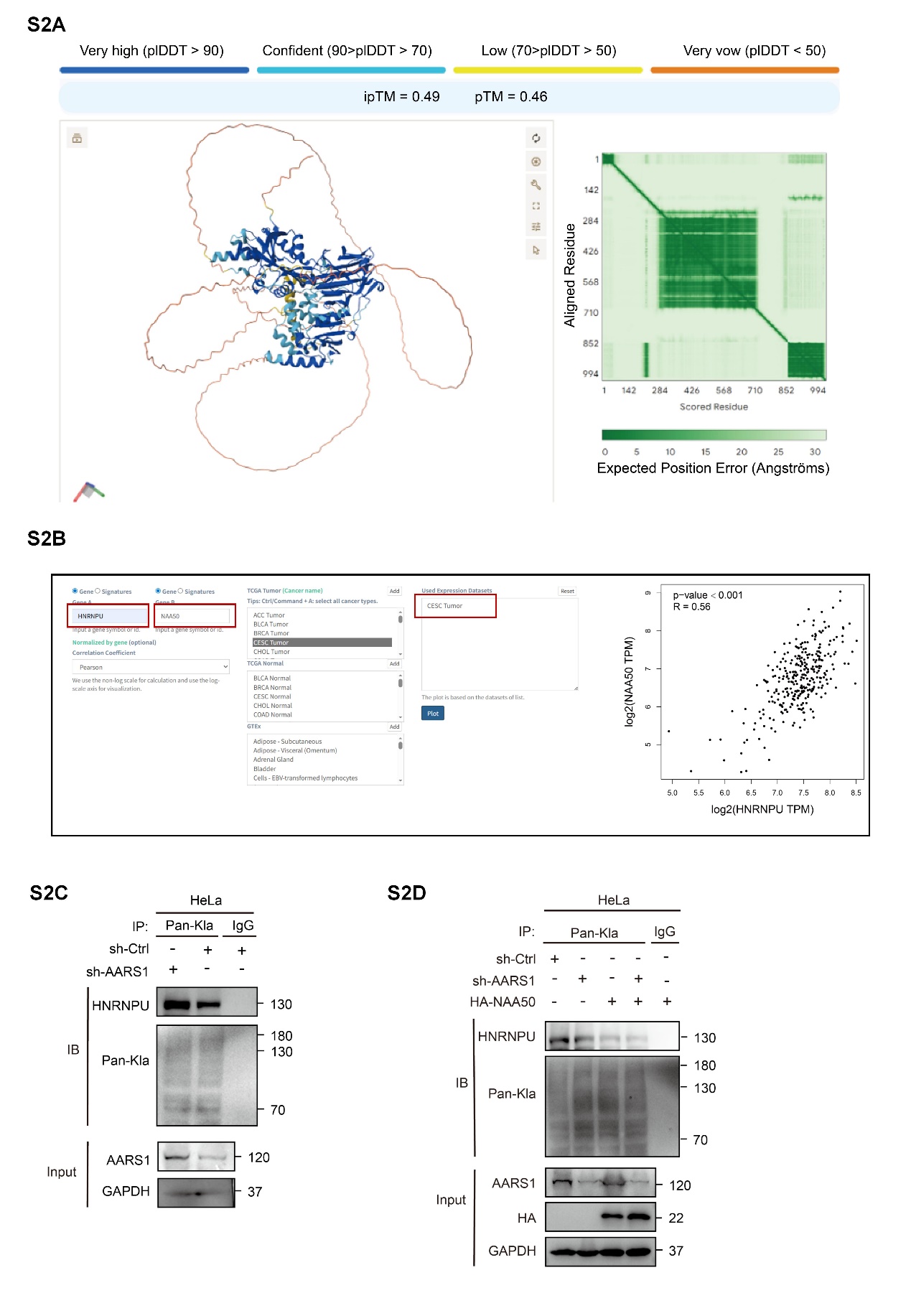


**Fig.S2 Structural prediction of the HNRNPU–NAA50 interaction using AlphaFold3.** A) Predicted three-dimensional structure of the HNRNPU (blue)–NAA50 (orange) complex, highlighting the high-confidence interaction interface (yellow). The structural model was generated using AlphaFold3 with default parameters. B) Scatter plots showing Pearson correlation analysis of HNRNPU and NAA50 mRNA expression (log_2_(TPM+1)) in TCGA cervical cancer (CESC) tumor samples (*R* = 0.56, *P* < 0.001). Analysis was performed using the GEPIA2 online tool (<http://gepia.cancer-pku.cn/>). C) HeLa cells were transfected with control shRNA (sh-Ctrl) or AARS1 shRNA (sh-AARS1). Cell lysates were immunoprecipitated with Pan-Kla antibody or control IgG, followed by immunoblotting (IB) with HNRNPU antibody to detect lactylated HNRNPU (top). Input lysates were analyzed by IB with AARS1 and GAPDH antibodies to confirm knockdown efficiency and equal loading. (Original uncropped blots can be found in Supplementary File 2, “Original blots for Fig. S2C.”) D) HeLa cells were transfected with the indicated shRNAs and/or HA-NAA50 overexpression plasmid. Lysates were immunoprecipitated with Pan-Kla antibody and immunoblotted with HNRNPU antibody (top). Input lysates were analyzed by IB with AARS1, HA (for NAA50), and GAPDH antibodies to confirm manipulation efficiency and equal loading (bottom). (Original uncropped blots can be found in Supplementary File 2, “Original blots for Fig. S2D.”)


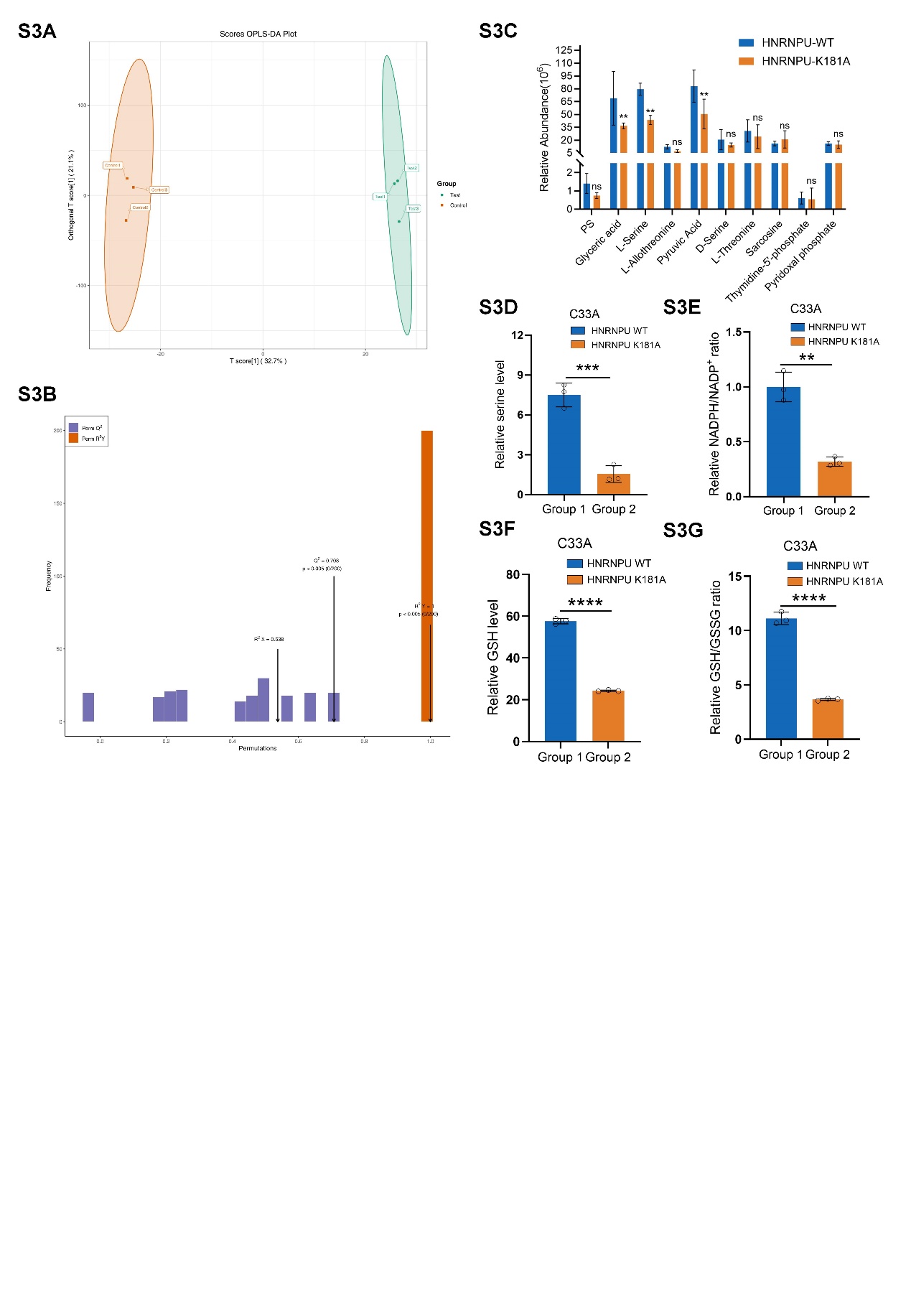
**Fig.S3 Validation of metabolomic profiling and serine metabolism alterations in C33A cells.** A) OPLS-DA score plot of metabolomic profiles from HNRNPU K181A mutant cells (Test, red) and wild-type controls (Control, blue). Each point represents an individual sample (n=3 per group). B) Permutation test of the OPLS-DA model with 200 random permutations. Model parameters: R²X=0.538, R²Y=1.00, Q²=0.708. The original Q² value (0.708, dashed line) is significantly higher than all permuted Q² values (*P*<0.005, 0/200), confirming model validity and excluding overfitting. C) Targeted quantification of key metabolites in the serine metabolic pathway (L-serine, glyceric acid, and pyruvic acid) in HNRNPU K181A mutant cells compared with controls, corresponding to Fig. 6C with additional technical replicates. Data are presented as mean ± SD from three independent biological replicates. Statistical significance was assessed using an unpaired two-tailed unpaired t-test. ***P* < 0.01. D-G) Functional validation of serine metabolism in C33A cells. The X-axis labels correspond to the following groups: Group 1, HNRNPU WT; Group 2, HNRNPU K181A. D) Intracellular serine levels; E) NADPH/NADP⁺ ratio; F) Total GSH content; G) GSH/GSSG ratio in HNRNPU K181A-expressing C33A cells versus controls. Data are mean ± SD from three independent biological replicates. Statistical significance was determined using a two-tailed unpaired t-test. ***P*<0.01, ****P*<0.001, *****P* <0.0001.


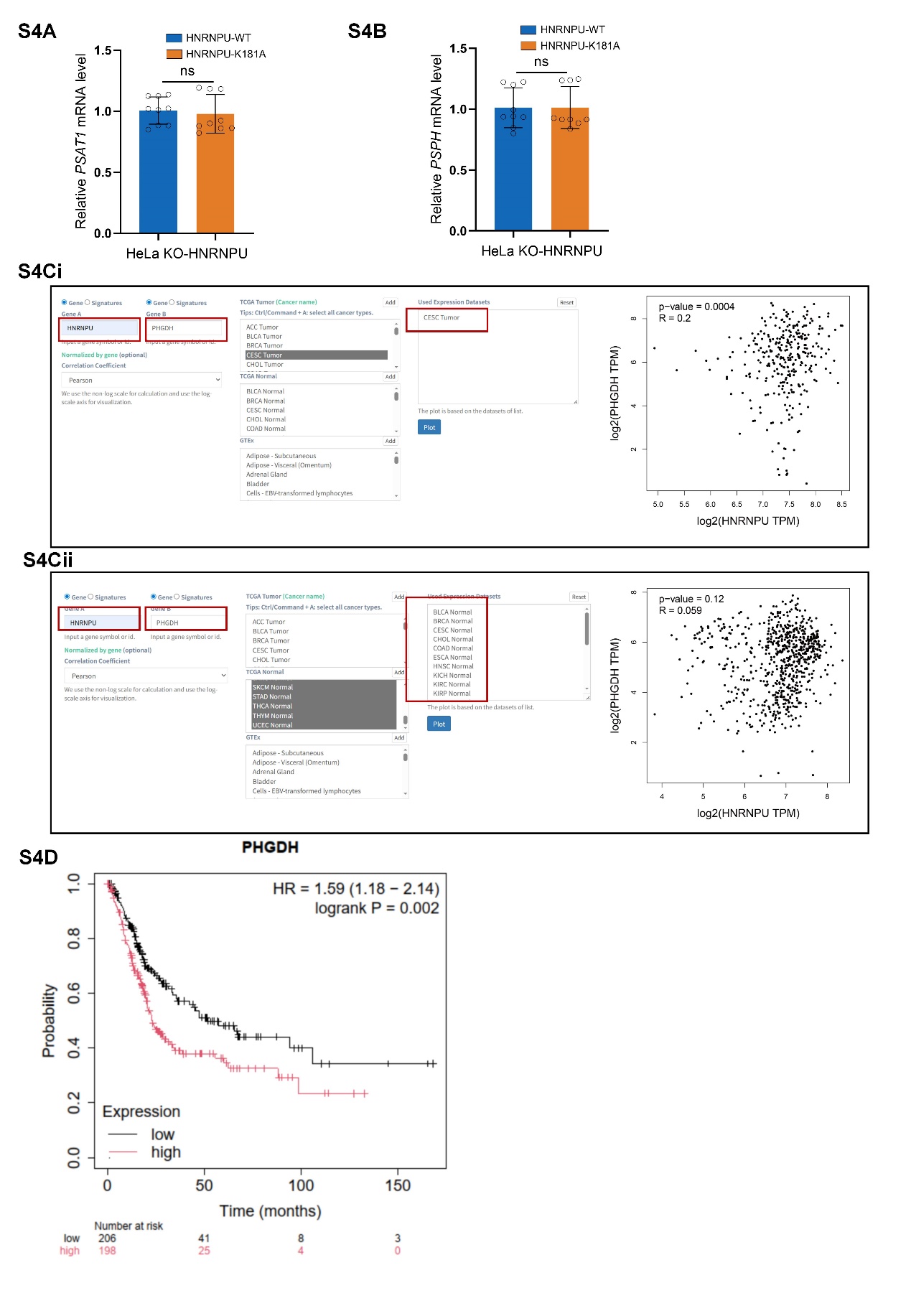


**Fig.S4 PHGDH is specifically regulated by HNRNPU K181 lactylation and correlates with cervical cancer prognosis.**  A, B) mRNA expression levels of PSAT1 and PSPH in HeLa KO-HNRNPU cells reconstituted with wild-type HNRNPU (HNRNPU-WT) or the lactylation-deficient mutant HNRNPU-K181A, as measured by qRT-PCR. Data are mean ± SD from three independent biological replicates. Statistical significance was determined using a two-tailed unpaired t-test. ns, not significant. C) Correlation analysis between HNRNPU and PHGDH expression in cervical cancer tumors from the TCGA‑CESC cohort (top) and in normal tissues from a large compendium (bottom). Pearson correlation coefficients (R) and *P*‑values are indicated. Analysis was performed using the GEPIA2 online tool (<http://gepia.cancer-pku.cn/> ). D) Kaplan‑Meier survival analysis showing the association between PHGDH expression and overall survival in cervical cancer patients. Patients were stratified into high‑ and low‑PHGDH expression groups based on the median expression value. Log‑rank test *P*‑value is shown. (KM Plotter; <https://www.kmplot.com>).


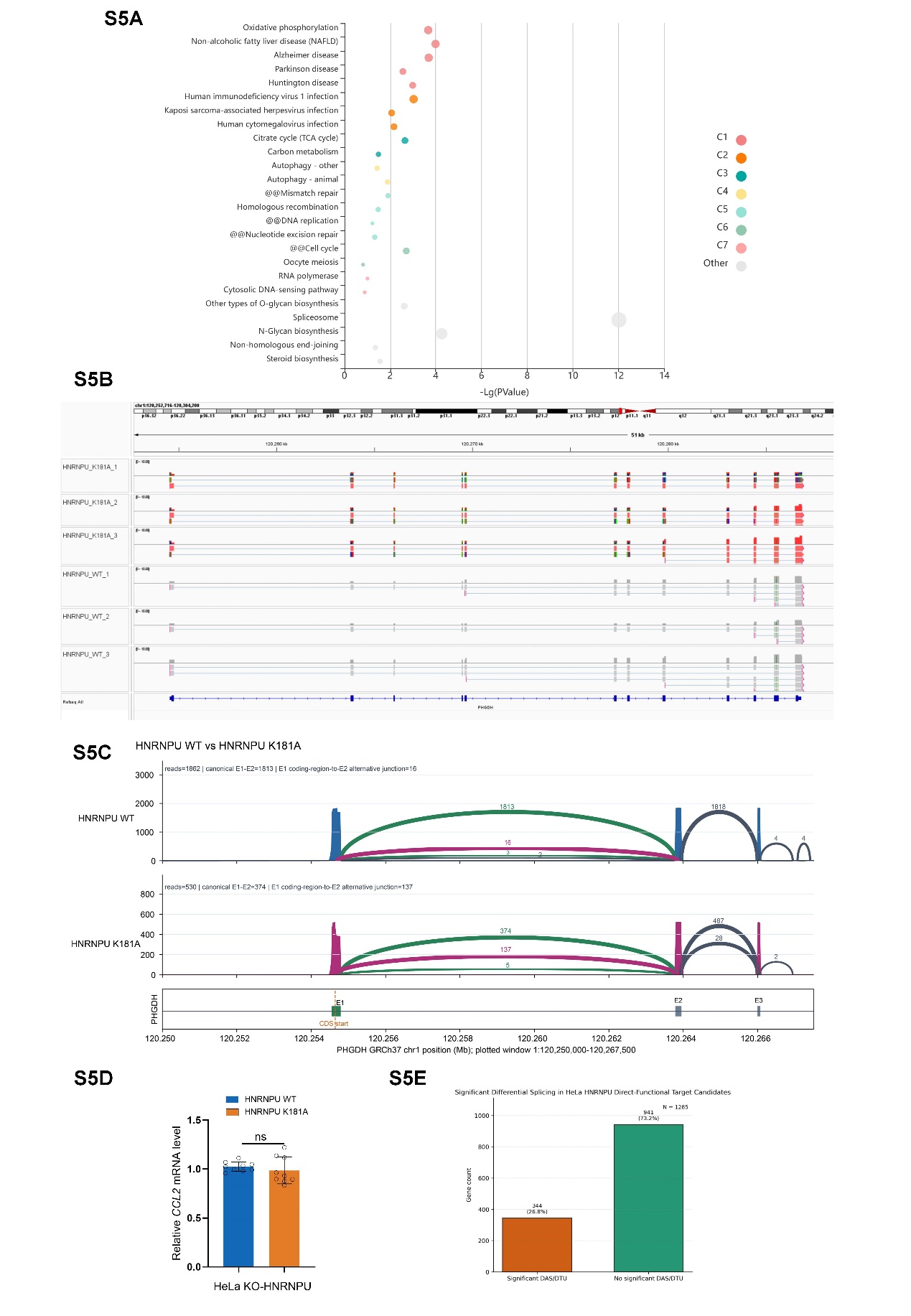
**Fig.S5 Transcriptome-wide splicing alterations and PHGDH-associated transcript analyses in HNRNPU K181A cells.** A) Pathway enrichment analysis of differential splicing genes derived from the most frequent SE (skipped exon) alternative splicing events, showing enrichment in carbon metabolism, tricarboxylic acid (TCA) cycle, spliceosome, and RNA polymerase-related pathways. B) Visualization of PHGDH read coverage and splice-junction patterns in HNRNPU K181A and WT cells. RNA-seq reads are displayed across the PHGDH genomic locus, with individual tracks representing biological replicates. Colored bars indicate mapped reads, and horizontal connecting lines represent splice-junction reads. In the K181A group, altered read distribution and splice-junction usage are observed at the 5′ region of PHGDH, particularly around the first exon-associated region. Notably, splice-junction signals connecting the exon 1-adjacent region to the downstream exon (exon 2) are visible in K181A samples, whereas such connections are less apparent in WT samples. Gene structure is shown at the bottom for reference. C) Sashimi plot of splice-junction usage between PHGDH exon 1 and exon 2 in HNRNPU WT and K181A cells. RNA-seq read coverage and splice-junction reads across PHGDH exon 1 and exon 2 are shown. Numbers above the arcs indicate junction-supporting reads. D) RT-qPCR analysis of CCL2 mRNA expression in WT and HNRNPU K181A cells. Relative mRNA levels were quantified and normalized to internal control genes. Each dot represents an independent biological replicate. No apparent difference in CCL2 mRNA expression is observed between WT and K181A groups under the same experimental conditions. E) Overlap analysis between HNRNPU direct-functional target candidates and significant DAS/DTU genes identified in the HNRNPU K181A dataset. Direct-functional targets were defined as genes both bound by HNRNPU (cyto-CLIP) and altered upon HNRNPU knockdown. The proportions of overlapping and non-overlapping genes are indicated.


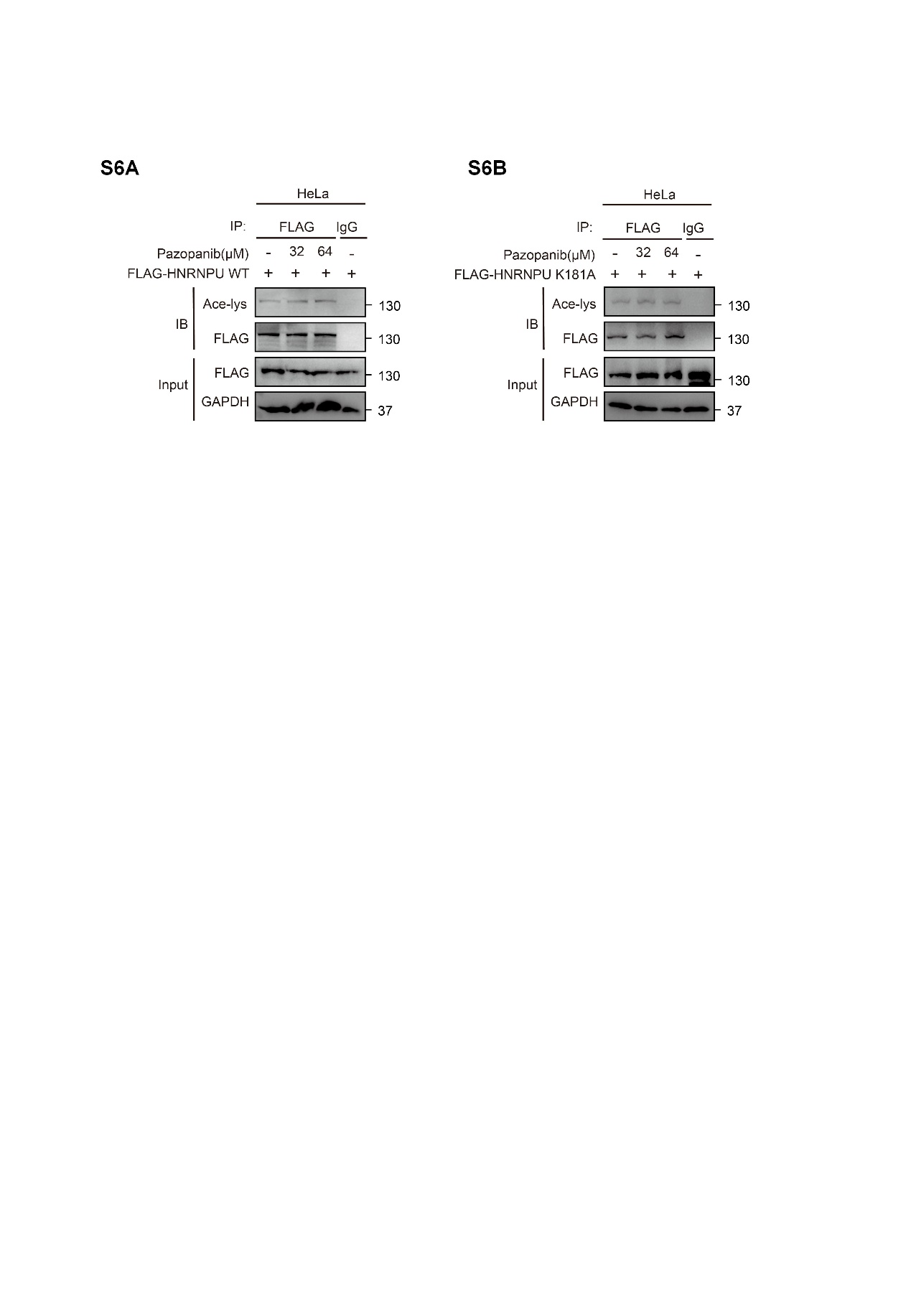


**Fig.S6 Western blot analysis of HNRNPU acetylation under Pazopanib treatment conditions.** A) Western blot analysis of HNRNPU acetylation in HeLa cells treated with Pazopanib (32 and 64 μM) under the same conditions as in Fig. 10H, I. (Original uncropped blots can be found in Supplementary File 2, “Original blots for Fig. S6A.”) B) Western blot analysis of HNRNPU acetylation in cells expressing the K181A mutant following Pazopanib treatment. (Original uncropped blots can be found in Supplementary File 2, “Original blots for Fig. S6B.”)

**Supplementary table 3**

| Gene Name | Sequence (5’-3’) |
| --- | --- |
| HNRNPU | F: TTCGTATTGGCTGGTCACTAACTAC |
|  | R: TCTCCATAATCTTCAGTCTCACAGTTG |
| PHGDH | F: CGCTGATGTCATCAACGCAG |
|  | R: TGGCCAGGCACATGATCATT |
| PSPH | F: TGTCAGAAATGACACGGCGA |
|  | R: GTAGGCGACTTACCAGCTCC |
| PSAT1 | F: TCCTTGTACAACACGCCTCC |
|  | R: GCGGCACCTCCATTGTTTTT |
| CCL2 | F: GACCATTGTGGCCAAGGAGA |
|  | R: TTGGGTTTGCTTGTCCAGGT |
